# Impact of Silver and Copper Oxide Nanoparticles on Anaerobic Digestion of Sludge and Bacterial Community Structure

**DOI:** 10.3390/nano15030236

**Published:** 2025-02-03

**Authors:** Zainab K. Abdulsada, Richard Kibbee, Juliska Princz, Banu Örmeci

**Affiliations:** 1 Department of Civil and Environmental Engineering, Carleton University, 1125 Colonel by Drive, Ottawa, ON K1S 5B6, Canada; zainababdulsada@cunet.carleton.ca (Z.K.A.); richardkibbee@cunet.carleton.ca (R.K.); 2 Environment and Climate Change Canada, 335 River Road South, Ottawa, ON K1V 1C7, Canada; juliska.princz@ec.gc.ca

**Keywords:** silver nanoparticles, copper oxide nanoparticles, sludge, anaerobic digestion, biogas generation, 16S rRNA gene sequencing

## Abstract

The effect of metal nanoparticles on the anaerobic digestion of sludge and the sludge bacterial community are still not well-understood, and both improvements and inhibitions have been reported. This study investigated the impact of 2, 10, and 30 mg/g TS silver and copper oxide nanoparticles (AgNPs and CuONPs) on the mesophilic anaerobic digestion of sludge and the bacterial community structure. The reactors were monitored for changes in tCOD, sCOD, TS, VS, biogas generation, and cell viability. Also, the relative abundance and taxonomic distribution of the bacterial communities were analyzed at the phylum and genus levels, including the genera involved in anaerobic digestion. Both AgNPs and CuONPs exhibited some inhibition on anaerobic digestion of sludge and biogas generation, and the inhibition was more evident at higher concentrations. CuONPs had a stronger inhibitory effect compared to AgNPs. After the introduction of AgNPs and CuONPs, cell viability initially decreased over the first two weeks but recovered after that. At high concentrations, AgNPs and CuONPs decreased the overall bacterial diversity, and inhibited the dominant bacterial species, allowing those in less abundance to flourish. The relative abundance of the bacteria responsible for hydrolysis and acidogenesis increased and the relative abundance of acetogenic bacteria decreased with higher AgNP and CuONP concentrations. The majority of the parameters measured for monitoring the anaerobic digestion performance and bacterial community were not statistically significant at 2 mg/g TS of AgNPs and CuONPs, which represents naturally present concentrations in wastewater sludge that are below the USEPA ceiling concentration limits.

## 1. Introduction

Anaerobic digestion is a widely used process for the treatment of wastewater sludge and converts biodegradable organic matter into energy through a series of complex microbial reactions. Successful anaerobic digestion depends on the types and critical functions of microbial communities that develop during digestion and are also influenced by sludge characteristics and the presence of contaminants such as metal nanoparticles.

AgNPs and CuONPs are known for their antimicrobial activity and have been widely used as preservatives or disinfectants in consumer products [1,2,3,4]. Their toxicity against a wide range of organisms and human cells has been documented [5,6,7,8]; potential mechanisms of toxicity include membrane disruption, DNA damage, enzyme inactivation, and apoptosis [9]. Due to their broad use, AgNPs and CuONPs are present in domestic wastewater in significant concentrations [10,11] and there are concerns that they can adversely affect biological wastewater treatment processes [12,13,14,15].

Liang et al. [16] investigated the impact of AgNPs on a modified Ludzack-Ettinger activated sludge system and observed a 46.5% decrease in denitrification when the system was exposed to 0.75 mg/L of AgNPs for 12 h. Sun et al. [17] showed that the presence of AgNPs at 1 mg/L for 24 h during the activated sludge process impacted some microbial species but did not decrease the heterotrophic plate count (HPC) before settling. After settling, the HPC decreased by one log with no observed changes in microbial diversity. Another study by Qiu et al. [18] found no significant effect of AgNPs on organic matter removal, nitrification, and denitrification during the sequencing batch reactor; however, increased production of extracellular polymeric substances due to cell self-protection was observed. Zhang et al. [19] studied the impact of CuONPs on a simulated waste-activated sludge system, and reported that the presence of 1, 10, and 50 mg CuONPs/L decreased the chemical oxygen demand (COD) removal from 78.7% to 77.0%, 52.1%, and 39.2%, respectively, and increased the effluent ammonium concentrations from 14.9 to 18.0, 25.1, and 30.8 mg/L, respectively.

Previous studies investigating the behavior and impact of AgNPs and CuONPs during anaerobic digestion demonstrated a decrease in biological activity [20,21,22]. For example, Rasool and Lee [22] observed decreases of 51.8 and 33.6% in sulfate and COD removal, respectively, at 50 to 200 mg/L concentration of AgNPs, but found no impact on sulfate and COD removal when using ≤10 mg/L AgNPs. Another study by Gonzalez-Estrella et al. [23] examined the inhibitory impacts of several different nanoparticles on methanogenic activity. They found that CuONPs inhibited acetoclastic methanogens (IC50 = 223 mg/L) without significant impacts on hydrogen-utilizing methanogens, while AgNPs also had no significant impact. Similar findings by Ünşar et al. [24] showed a 5.8 to 84% inhibition of anaerobic digestion when using 5–1000 mg/g total solids (TS) of CuONPs. AgNPs did not show a similar inhibition on anaerobic digestion but decreased methane production by 12.1% at the highest dose of 1000 mg/g TS.

Although several studies suggest nanoparticles lower the volume of methane collected [20,24,25], there are other studies that have reported improvements to anaerobic digestion and biogas production [9,26]. The reported improvements were mainly attributed to direct interspecies electron transfer and mostly observed for FeNP, while mixed results were reported for other NPs including AgNP, but no studies reported improvements with CuONP [9]. These findings indicate that the effect of NPs on the anaerobic digestion process itself and the microorganisms involved are still not well-understood.

This study investigated the impact of both low (2 mg/g TS of sludge) and high (10 and 30 mg/g TS of sludge) concentrations of AgNPs and CuONPs on mesophilic anaerobic digestion of sludge with a comprehensive evaluation of the changes that occur in bacterial community structure using 16S rRNA gene sequencing. There are currently no limits on Ag in treated sludge (biosolids) in the US (USEPA 40 CFR Part 503). The ceiling concentration limit for Cu is 4300 mg/kg DS (TS), which is more than twice the concentration (2 mg/g TS of sludge, equivalent to 2000 mg/kg TS) used in this study. In addition to the environmentally relevant concentrations, high concentrations of AgNPs and CuONPs were included in the study to better understand the extent and impact of toxicity. The objectives of this study were to 1. Investigate the effect of AgNPs and CuONPs on the anaerobic digestion of sludge and biogas generation, 2. Investigate the effect of AgNPs and CuONPs on the bacterial community structure, diversity, abundance, and functions, 3. Explore the effect of exposure to increasing concentrations of AgNP and CuONP, and 4. Determine whether there would be significant concerns to biological treatment at concentrations naturally present in sludge.

## 2. Materials and Methods

### 2.1. Silver and Copper Oxide Nanoparticles

The stock suspension of pristine AgNPs in water with a purity of 99.9% was acquired from nanoComposix Inc. (San Diego, CA, USA) and maintained at 4 ± 0.2 °C. The stock solution of CuONPs with 99.95% purity was purchased from the U.S. Research Nanomaterials Inc., Houston, TX, USA, and maintained at 4 ± 0.2 °C. Both nanoparticles’ suspensions were stable and insoluble in nanopure water, and their particle size distributions were confirmed by transmission electron microscopy (TEM)(FEI Tecnai G2 F20, FEI, Eindhoven, The Netherlands) at our Nano Imaging facility at Carleton University, Ottawa, Canada. The properties of both nanoparticles are shown in Table 1.

### 2.2. Wastewater Sludge Samples

Three types of wastewater sludge were collected from a conventional activated sludge treatment plant in Ontario, Canada. Primary sludge was obtained from the primary clarifiers, thickened waste activated sludge (tWAS) was collected after centrifuge thickening, and anaerobically digested sludge was collected from the mesophilic (35 °C) anaerobic digesters. The anaerobically digested sludge was used as an inoculum for the anaerobic digestion of the mixed primary and tWAS. All wastewater sludge samples were collected in autoclaved containers and were used either on the day of collection or within 4 days after storing at 4 °C. The characteristics of the primary, tWAS, and anaerobically digested sludge are provided in Table 2.

### 2.3. Experimental Design

Anaerobic digestion was carried out using batch BMP tests conducted in 125 mL Erlenmeyer flasks sealed with rubber stoppers. There were seven BMP reactors, of which one reactor was the control with no nanoparticles, three reactors had increasing concentrations of AgNPs (2, 10, and 30 mg AgNPs/g TS), and three reactors had increasing concentrations of CuONPs (2, 10, and 30 mg CuONPs/g TS). All tests were performed in triplicate. A total of 2 mg NPs/g TS was chosen to represent the higher end of the reported nanoparticle concentrations in wastewater sludge [27,28,29,30], and 10 and 30 mg NPs/g TS were selected to observe the impact of higher NP concentrations [21]. Table 3 shows the concentration and experimental conditions of the reactors. Each Erlenmeyer flask reactor contained 15 mL of anaerobically digested sludge (inoculum), 35 mL of primary sludge and 35 mL of tWAS, with a final sludge volume of 85 mL. In addition, equal amounts of NaHCO_3_ and KHCO_3_ were added to achieve an alkalinity of 4000–5000 mg/L (as CaCO_3_). In order to achieve a substrate to inoculum ratio (S/I) of 1.5, the anaerobically digested sludge was first thickened with centrifugation at 1800 rpm for 10 min to increase its volatile solids. Then, the three concentrations of AgNPs or CuONPs were added to the BMP reactors (except the control) as described in Table 3. During these steps, BMP reactors were mixed continuously with nitrogen sparging to prevent exposure to oxygen in ambient air and provide mixing. The reactors were kept in the dark, at 34 ± 1 °C, and mixed on a rotating shaker at 100 rpm in a temperature-controlled incubator for 37 days. Reactors were sampled periodically for sludge characterization and microbiological tests including biogas volume, TS, VS, COD, live/dead bacterial viability staining, and DNA extractions for population sequencing.

### 2.4. Chemical Analyses

Chemical analyses of sludge from each reactor were carried out in triplicate on days 0, 4, 9, 13, 17, 26, and 37 following Standard Methods [31], and means and standard deviations were calculated. COD was measured at a wavelength of 620 nm with a HACH DR2800 spectrophotometer using the HACH Method 8000 [31]. Soluble COD measurements were performed on the filtrate of the sludge samples after centrifugation at 1800 rpm for 20 min, followed by filtration of the supernatant through a 0.45 µm syringe filter (Glassco Laboratory Equipment, Ambala Cantt, India). TS and VS were determined based on the Standard Methods, Part 2540 [31]. The pH was monitored using an Orion Star _TM_ A326 pH/DO portable meter (ThermoFisher Scientific, Waltham, MA, USA).

### 2.5. Biological Analyses

There were six time point measurements for each reactor over the duration of the experiments (days 0, 4, 9, 17, 26, and 37).

#### 2.5.1. Live/Dead Bacterial Viability Staining Assay

Live/dead bacterial viability staining assay was used to distinguish viable and nonviable cells in the microbial population. Sludge samples were stained as per instructions described in the BacLight^®^ Live/Dead viability assay kit (Molecular Probes, Eugene, OR, USA), and viewed using a Nikon Eclipse Ti (Nikon, Montreal, QC, Canada) inverted fluorescence microscope. Three tests were performed for each sludge sample, and three random fields of view (FOV) were chosen on the slide. Three images were taken at each FOV using three UV filters (GFP-1, Cy-3 and GFPHQ) to capture live, dead, and a combination of live and dead cells, respectively. The three sets of images were assessed, and the most representative was chosen to report. The images were captured with the Q-Imaging Retina Exi Fast 1394 camera (Nikon, QC, Canada) and the image analysis was performed with NIS-Elements AR software version 5.42.01 (Nikon, QC, Canada).

#### 2.5.2. DNA Extraction and Quantification

Genomic DNA was extracted from the reactor samples using a DNeasy PowerSoil DNA Isolation Kit (QIAGEN Inc., Hilden, Germany). To help reduce qPCR inhibitors and remove extracellular DNA, all sludge samples were prewashed according to Kibbee and Örmeci [32] before the extraction. Three replicate DNA extractions were performed for each sludge sample according to the manufacturers’ instructions, with the exception of the cell lysis and inhibition removal steps where the incubation times were doubled from five to ten minutes. The concentration of extracted DNA was measured using the Qubit^®^ dsDNA HS Assay Kit with a Qubit 3.0 Fluorometer from Molecular Probes (Life Technology, Eugene, OR, USA). The DNA purity was assessed by measuring the absorbance ratios at 260/280 and 260/230 nm using NanoDrop 1000 Spectrophotometer (Thermo Scientific, Wilmington, NC, USA). The genomic DNA was also assessed using gel electrophoresis to ensure fragment sizes were between 3 and 10 Kbps, as required for optimal MinION sequencing. Genomic DNA extracts were run on a 0.8% agarose gel at 7 V/cm^2^; a TAE buffer was used to prepare the agarose gel. 6X gel loading dye and GeneRuler 1 Kb ready-to-use DNA Ladder (Thermo Fisher) were used to visualize the genomic DNA fragment size range. The DNA samples were stored at −20 °C until use.

#### 2.5.3. High-Throughput Sequencing/Bacterial Community Analyses

Bacterial diversity and population profiles of the sludge samples were analyzed using the 16S rRNA BLAST results generated from the MinION sequencing runs and EPI2ME workflows. Seven genomic DNA libraries, prepared according to the MinION 1D 16S Barcoding Kit (SQK-RAB204) protocol, were used to generate 16S bacterial community profiles. Each library consisted of 6 uniquely barcoded time points run separately in a MIN 107 MinION flowcell. The base-called data from each run was then used in the EPI2ME Fastq 16S workflow. This workflow generated BLAST data, which was used directly for bacterial diversity and community analyses. The MinION device, flowcell, and kits were purchased from Oxford Nanopore Technologies (New York, NY, USA), and the library preparation and sequencing were performed according to the MinION 1D 16S Barcoding Kit (SQK-RAB204) protocol. The DNA samples were amplified by PCR using the kit-specific 16S primers (27F and 1492R) with barcodes and 5′ tags that facilitate ligase-free attachment of Rapid Sequencing Adapters. The PCR products were cleaned and concentrated with Agencourt AMPure XP beads (Beckman Coulter, Mississauga, Canada) and quantified with the Qubit 3.0. The PCR products for each library were then pooled at the required ratio, rapid sequencing adapter attached, loading beads added, and then loaded into the MinION flowcell. Using the MinKNOW software Version 1.15.4 (Oxford Nanopore Technologies) with real-time base-call monitoring, the sequencing was stopped at ~150,000 reads to maintain a consistent read depth for all reactor samples.

#### 2.5.4. 16S rRNA Gene Sequencing

The MinKNOW generated Fastq files were BLASTed against the NCBI 16S bacterial database, using Oxford Nanopore’s cloud-based analysis platform EPI2ME with the 16S Fastq workflow. Each read was classified based on % coverage and identity. Results were transcribed into Excel data sheets where they were sorted into different taxonomy classifications (phylum and genus level) for every time point and BMP reactor. A 3% sequence dissimilarity cut-off value, commonly used to estimate the species level of phylotype diversity, was universally applied. During the study, biodiversity was estimated for the top detected phyla with a relative abundance of ≥ 0.01%. Alpha biodiversity (Shannon-Wiener index, evenness, and richness) and Beta-Euclidean biodiversity were estimated and analyzed for bacteria at the phylum level. Relative abundance was estimated and compared for each BMP reactor at phylum and genus levels. The cutoff value for biodiversity analysis at the genus level was ≥ 1% relative abundance. Several interesting genera were discussed, including those with a relative abundance of ≤1%. These genera were selected based on their function in the anaerobic digestion process, as cited in the literature, and shown in Table S1 in the Supplementary Materials. The Shannon-Wiener index, used to characterize the diversity of phyla in a community, was determined by calculating the percentage of a phylum and comparing it to the other phyla in a community, then multiplying it by the natural logarithm of this proportion. The resulting values are summed across the phyla and multiplied by −1 Equation (1) [33]. Evenness was calculated according to Equation (2) [33,34]. Evenness is between 0 and 1, with 1 being complete evenness. Richness (N) is the total number of phyla after the cut-off.

(1) $$H=-\sum_{i=1}^{N}pi\ln pi$$

(2) $$E=\frac{H}{Hmax}=H/\ln N$$

where

*H*: Shannon-Wiener index
*N*: total number of phyla
*pi*: proportion of phylum *i* to all other phyla
*E*: phyla evenness

Beta-Euclidean diversity was calculated according to Equation (3) [35].

(3) $$Beta-Euclidean=sqrt(\sum_{1}^{N}{\left(nki-nkj\right)}^{2})$$

where

*N*: total number of phyla
*nki*: count of OTU *k* in sample *i*
*nkj*: count of OTU *k* in sample *j*

### 2.6. Statistical Analyses

A one-way analysis of variation (ANOVA) at a 95% confidence level was used to study and evaluate significant differences between the reactors over time; the difference was considered significant if *p*-values were <0.05. If there were significant differences between two reactors, the post hoc Tukey test was used to further separate mean values, using the same significance level [36]. Three replicates were performed for each analysis, and the results are reported with average ± standard deviations. To compare values within the same group, the coefficient of variance (CV) was applied to compare an individual data set, if the CV is ≤ 0.05 then the data are not significantly different (i.e., the confidence is ≥95%).

## 3. Results and Discussion

### 3.1. Anaerobic Digestion Performance

**Figure 1:**
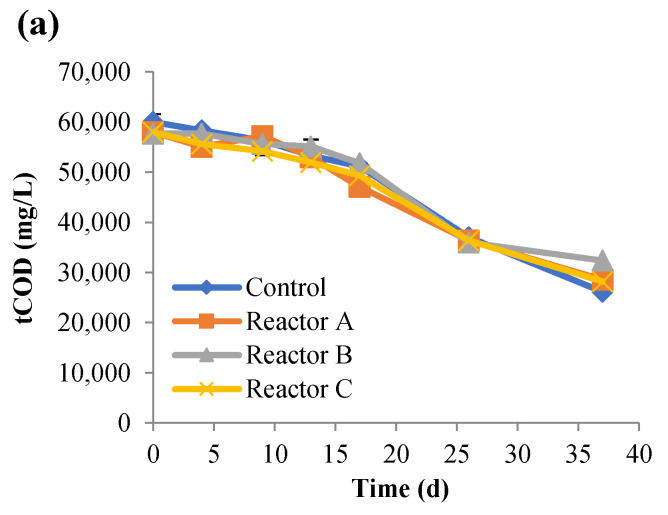

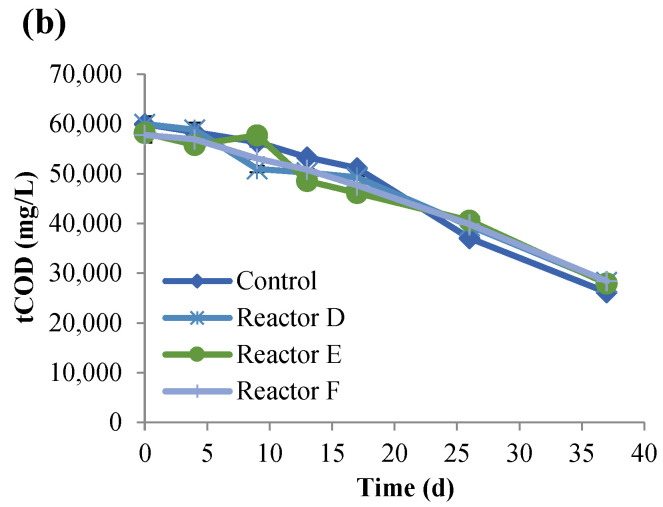
Concentrations of tCOD over time, for the control and reactors A, B, and C (**a**), and reactors D, E, and F (**b**) (the control had no nanoparticles and reactors A, B, and C had 2, 10, and 30 mg AgNPs/g TS, respectively, and reactors D, E, and F had 2, 10, and 30 mg CuONPs/g TS, respectively).

**Figure 2:**
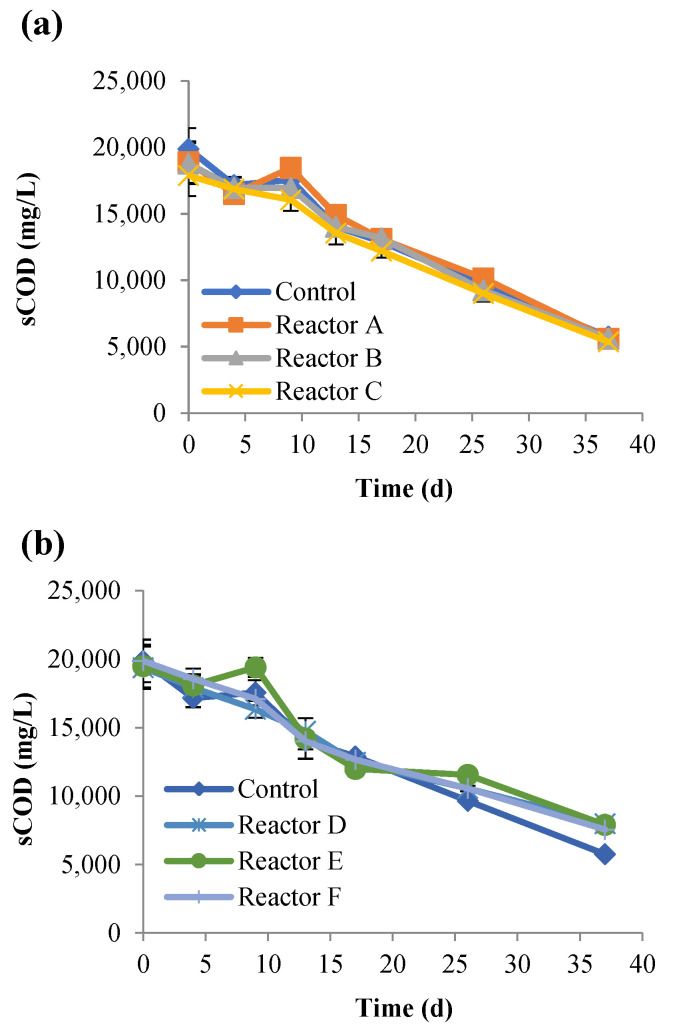
Concentrations of sCOD over time, for the control and reactors A, B, and C (**a**), and reactors D, E, and F (**b**) (the control had no nanoparticles and reactors A, B, and C had 2, 10, and 30 mg AgNPs/g TS, respectively, and reactors D, E, and F had 2, 10, and 30 mg CuONPs/g TS, respectively).

The progression of anaerobic digestion was examined by monitoring the changes in the total and soluble COD (tCOD and sCOD), TS, VS, and biogas generation. The concentrations of tCOD and sCOD (Figure 1 and Figure 2) steadily decreased over time with no significant variations between the control and the other reactors (*p*-values > 0.05). There were no significant differences in the percentage removal of tCOD between the AgNP and CuONP reactors, but they were slightly lower (approximately 5%) compared to the control reactor. Percent sCOD removals were approximately 10% lower in the CuONP reactors compared to the control and the AgNP reactors (Table 4) with no significant differences, except for reactor F (*p* value = 0.01).

**Figure 3:**
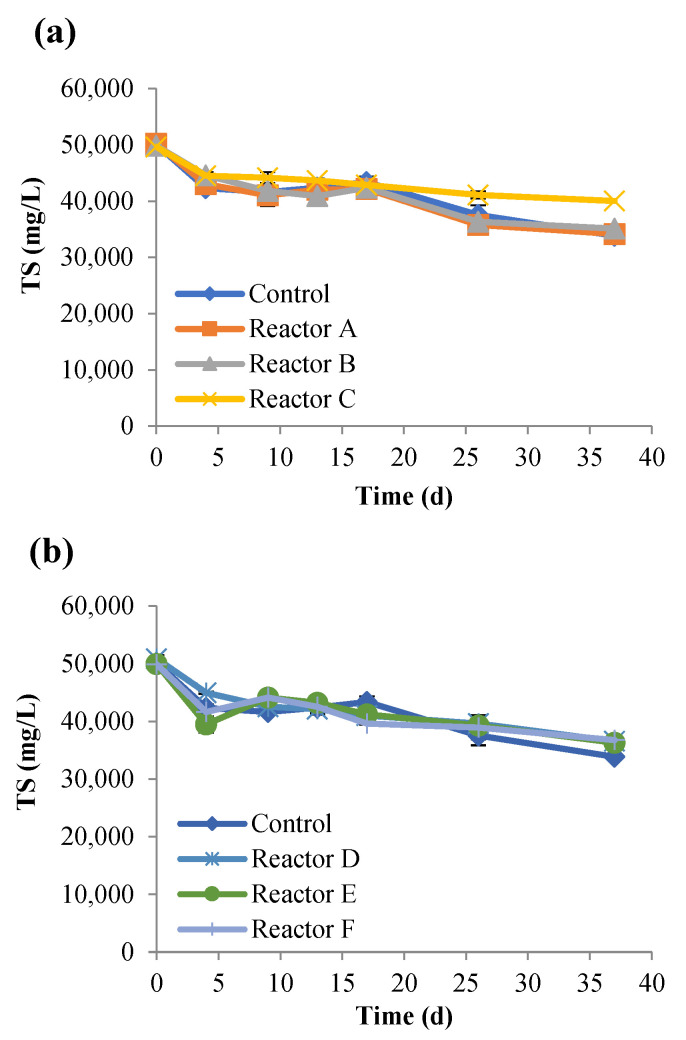
Concentrations of TS over time, for the control and reactors A, B, and C (**a**), and reactors D, E, and F (**b**) (the control had no nanoparticles and reactors A, B, and C had 2, 10, and 30 mg AgNPs/g TS, respectively, and reactors D, E, and F had 2, 10, and 30 mg CuONPs/g TS, respectively).

**Figure 4:**
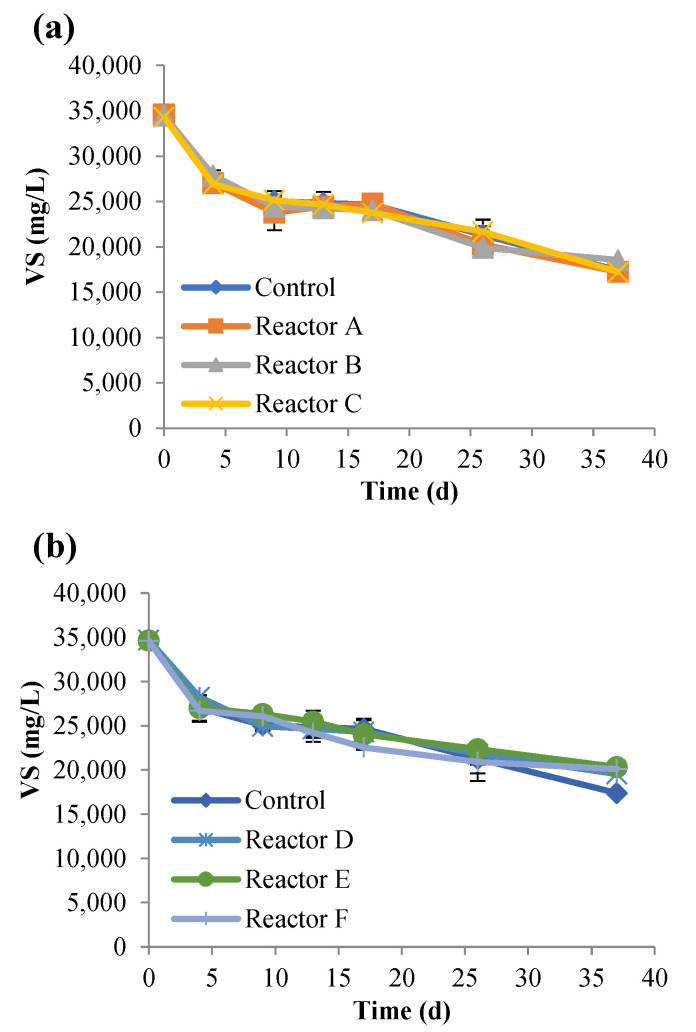
Concentrations of VS over time, for the control and reactors A, B, and C (**a**), and reactors D, E, and F (**b**) (the control had no nanoparticles and reactors A, B, and C had 2, 10, and 30 mg AgNPs/g TS, respectively, and reactors D, E, and F had 2, 10, and 30 mg CuONPs/g TS, respectively).

**Figure 5:**
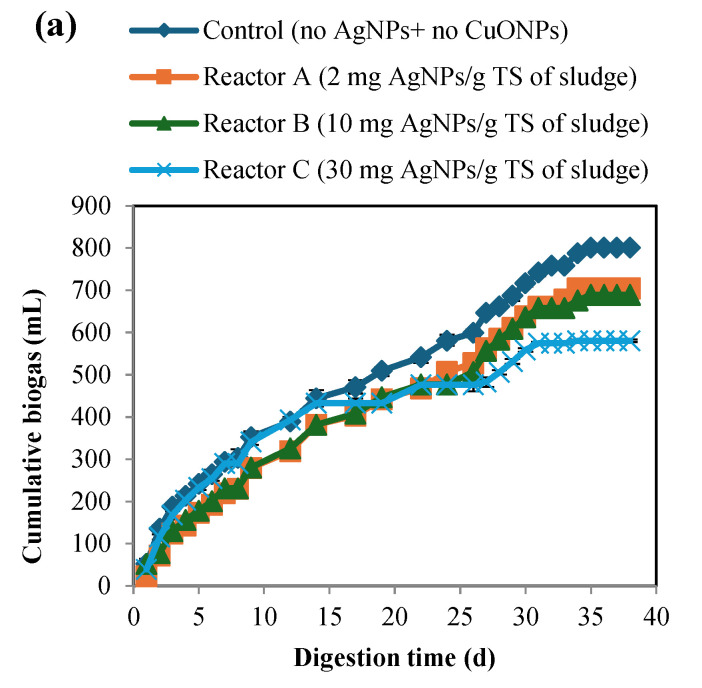

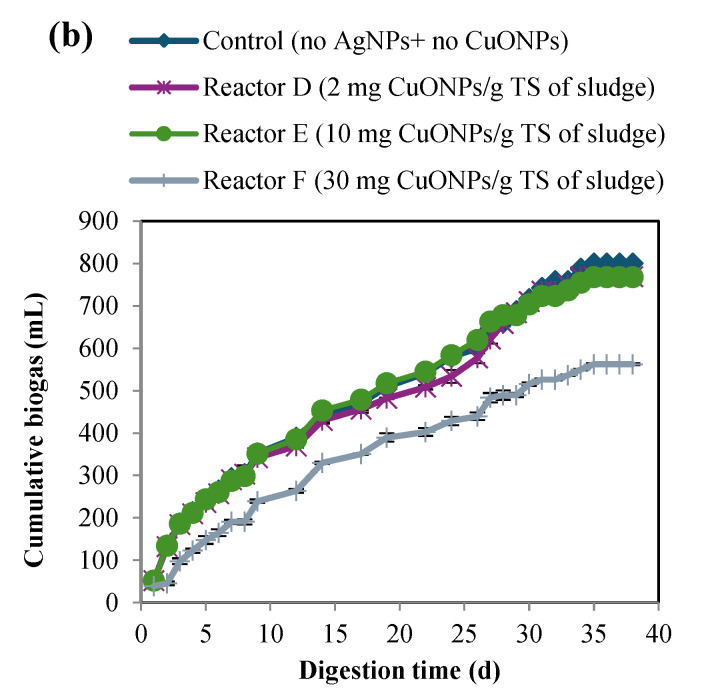
Cumulative biogas production over time for BMP reactors that had AgNPs (**a**) and CuONPs (**b**) with negative controls for each.

The changes in TS and VS were also investigated (Figure 3 and Figure 4), and the results demonstrated that both TS and VS decreased gradually over time and showed similar trends at increasing concentrations of AgNPs and CuONPs. As shown in Table 4, the percent removal of VS was lower in the CuONP reactors compared to the control (*p*-value was 0.03 for reactor F compared to control), but there were no significant differences among the AgNP and CuONP reactors (*p* > 0.05). Biogas generation also decreased with increasing concentrations of AgNPs and CuONPs (Figure 5). Based on ANOVA analysis, reactors C and F were significantly different than the control (*p*-values were 0.11, 0.09, 0.03, 0.4, 0.45, and 0.003 for reactors A, B, C, D, E, and F, respectively).

Previously, Hou et al. [37] reported that AgNPs did not significantly impact the removal of COD during sequencing batch reactor treatment of wastewater, which is an aerobic process. On the impact of AgNPs on anaerobic digestion, previous studies reported inhibition [21,38] as well as no significant impact [39,40]. The inhibitory impact of CuONPs on anaerobic digestion appears to be higher than that for AgNPs, which was also reported by other studies. For example, Luna-delRisco et al. [20] demonstrated that the presence of CuONPs at 10.7 mg Cu/L decreased methane production by 50% during anaerobic digestion of cattle manure for 14 d at 35 °C. Another study by Otero-González et al. [41] found that long-term exposure (126–150 d) to CuONPs at 1.4 mg Cu/L during anaerobic digestion of synthetic wastewater at 30 ± 2 °C reduced methane production by more than 50% due to severe toxicity that reduced more than 85% of the acetoclastic methanogenic activity. Gonzalez-Estrella et al. [23] and Ünşar et al. [24] reported similar results.

### 3.2. Impact of AgNPs and CuONPs on Cell Viability

**Figure 6:**
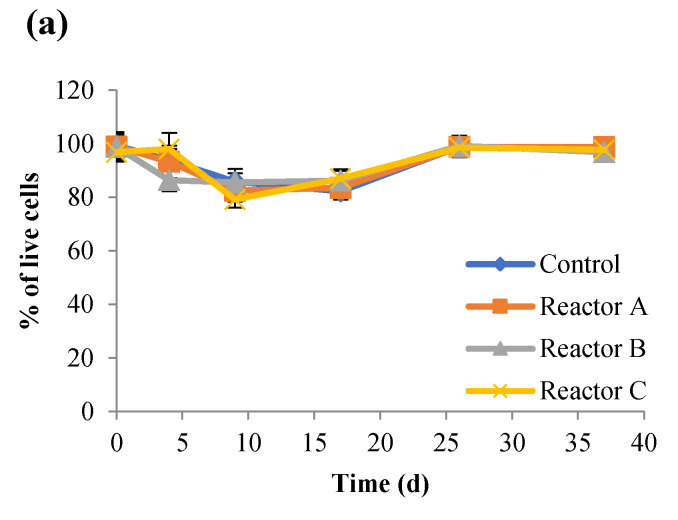

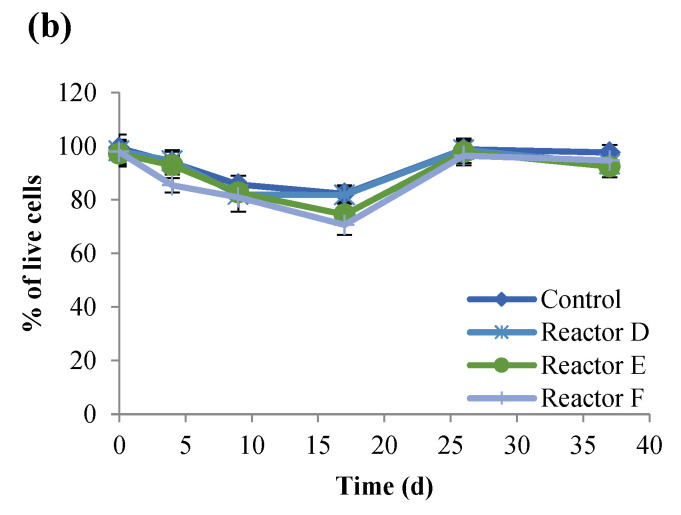
Percent live cells for the control and reactors A, B, and C (**a**), and reactors D, E, and F (**b**) (the control had no nanoparticles and reactors A, B, and C had 2, 10, and 30 mg AgNPs/g TS, respectively, and reactors D, E, and F had 2, 10, and 30 mg CuONPs/g TS, respectively).

**Figure 7:**
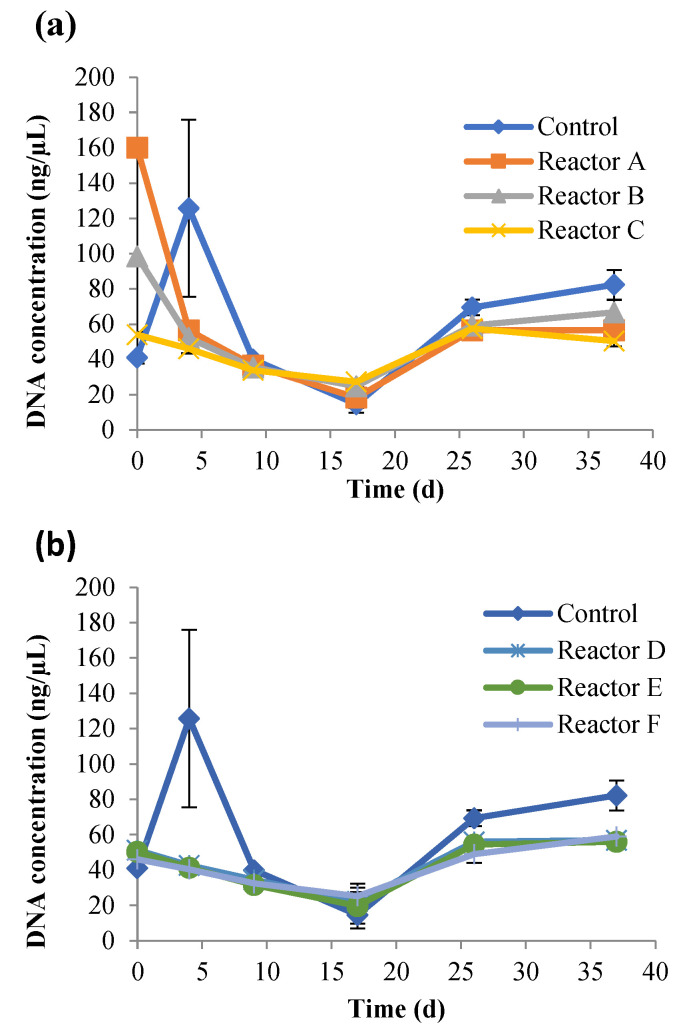
Total genomic DNA over time, for the control, and reactors A, B, and C (**a**), and reactors D, E, and F (**b**) (the control had no nanoparticles and reactors A, B, and C had 2, 10, and 30 mg AgNPs/g TS, respectively, and reactors D, E, and F had 2, 10, and 30 mg CuONPs/g TS, respectively).

The impact of AgNPs and CuONPs on the viability of sludge bacteria was investigated using a Live/Dead viability assay. Viable cells with intact membranes are stained and fluoresce green, while cells with compromised membranes are considered non-viable, and are stained and fluoresce red under the microscope. Results of live cell percentages in sludge reactors are shown in Figure 6, and representative Live/Dead cell images at each time point for each reactor are shown in Figures S1–S7 (Supplementary Materials). The percentage of live cells showed a similar trend for the control and test reactors over time with reactors E and F having the lowest live cell percentages, with no statistically significant differences (*p* > 0.05) between the control and other reactors. Overall, the results showed that AgNPs had no significant impact on the live cells while CuONPs decreased the percentage of the live cells. As seen in Figure 6, the percentage of live cells decreased in all reactors until day 17, when the percentage of live cells started to increase. This was supported by the DNA concentrations over time (Figure 7), and may be due to a compensatory effect. Kaweeteerawat et al. [42] reported that AgNPs inhibited *Escherichia coli* and caused oxidative stress, which eventually led to increased bacterial resistance to antibiotics due to increased stress tolerance. Prolonged exposure to AgNPs can alter the cell membrane lipids [43] and cause physiological changes in bacteria resulting in antibiotic resistance.

### 3.3. Impact of AgNPs and CuONPs on Bacterial Community Structure

**Figure 8:**
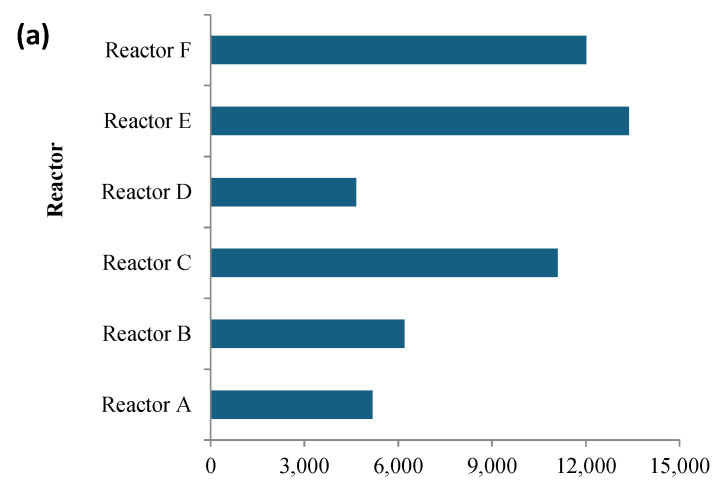

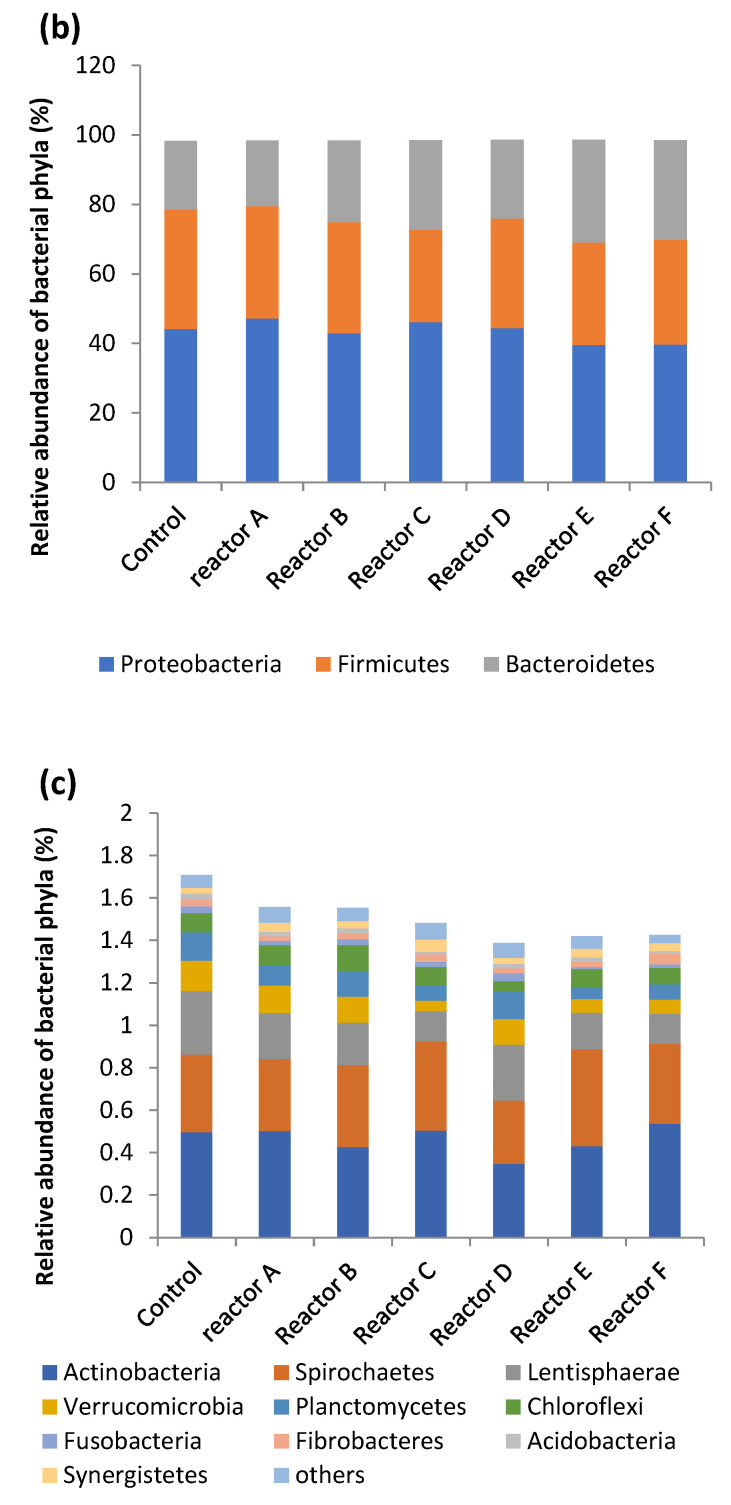
Beta-Euclidean diversity (**a**), the most abundant phyla ≥98% of the observed OTUs (**b**), and the next most abundant phyla ≤2% of the observed OTUs (**c**). Phyla with ≤0.01% relative abundance were not included. (Reactors A, B, and C contain 2, 10, and 30 mg AgNPs/g TS, respectively, and reactors D, E, and F contain 2, 10, and 30 mg CuONPs/g TS, respectively).

The relative abundance and taxonomic distribution of the bacterial community in each DNA extraction (DNA concentrations shown in Figure 7) were analyzed at the phylum and genus levels, including the genera related to functions in the anaerobic digestion process. The sequencing results indicated that each sludge reactor had highly diverse bacterial communities (Figure 8). The number of species represented by unique operational taxonomic units (OTUs) tallied from all time points, were 2157, 2192, 2148, 2117, 2151, 1925, and 2045, for the control and reactors A, B, C, D, E, and F, respectively. It is evident that AgNPs and CuONPs at high concentrations decreased the overall species richness in these reactors.

**Figure 9:**
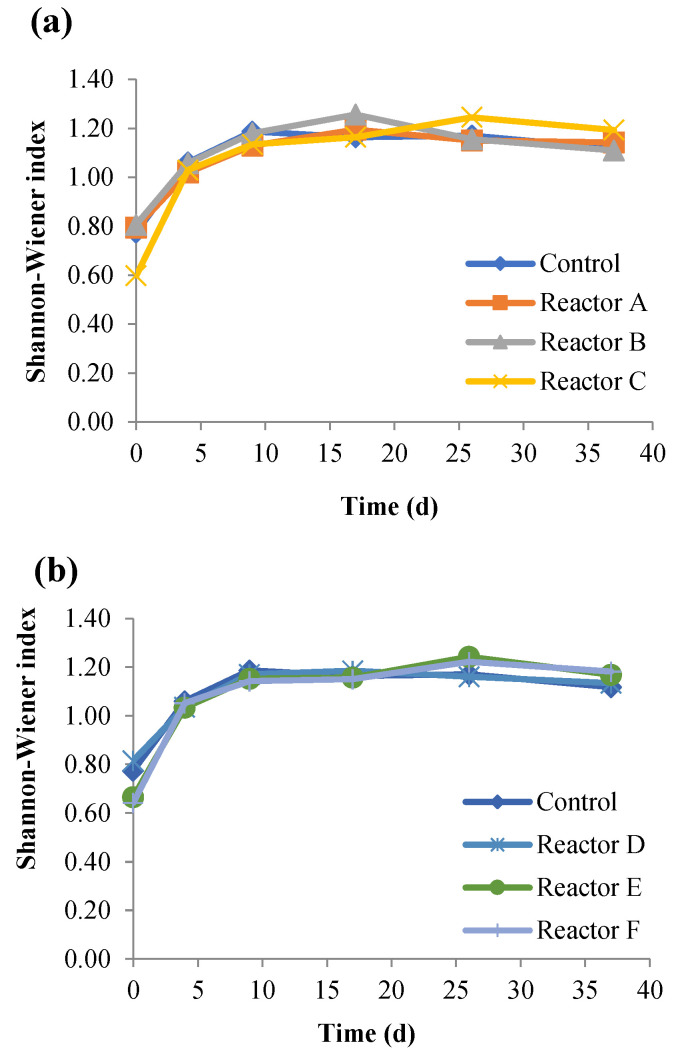
Shannon-Wiener index comparing number of species per phyla for the control and reactors A, B, and C (**a**), and reactors D, E, and F (**b**) (the control contains no nanoparticles and reactors A, B, and C contain 2, 10, and 30 mg AgNPs/g TS, respectively, and reactors D, E, and F contain 2, 10, and 30 mg CuONPs/g TS, respectively).

Bacterial Alpha and Beta-Euclidean biodiversity was estimated at the phylum level. Alpha diversity is a standard measure of the phyla/genera/species diversity in a community, while Beta-Euclidean diversity is a measure used to compare the composition between two or more communities. Alpha diversity is represented by the Shannon-Wiener index (characterizes the composition and commonness of bacteria in a community), evenness (measures how close in number each bacterial taxonomic group in a community is), and richness (the number of unique OTUs or phyla/genera/species in a community). The estimated Shannon-Wiener index and the different phyla presented, as shown in Table 5, were in most cases slightly higher in the reactors with higher concentrations of nanoparticles. Similar observations were reported by Huang et al. [44], who showed an increase in the Shannon-Wiener index with higher doses of tetracycline. It is possible that the antimicrobial properties of nanoparticles adversely impact the growth of the dominant bacteria (most abundant bacteria) in sludge allowing less abundant bacteria to thrive, resulting in an increased Shannon-Wiener index and the number of different phyla presented [44,45,46]. Another explanation is that these species could be nanoparticle resistant bacteria. For example, Huang et al. [47] showed that the presence of CuONPs at 20 mg/g total suspended solids (TSS) during anaerobic digestion increased the abundance of antibiotic-resistant genes with no significant impact on the resistance mechanisms and types. The Shannon-Wiener index for each reactor over time was also assessed (Figure 9), and the results showed no significant differences (*p*-values ˃ 0.05). The bacterial diversity represented by the Shannon-Wiener index was low for each reactor on day 0, particularly in reactors C, E, and F, which were lower than the control by 26.0, 18.5, and 3.5%, respectively. This may be due to the toxicity of higher concentrations of AgNPs (30 mg/g) and CuONPs (10 and 30 mg/g) in those three reactors. The Shannon-Wiener index increased significantly for all reactors in the first 4–9 days and then stabilized for the duration of the experiment (*p*-value = 0.99).

**Figure 10:**
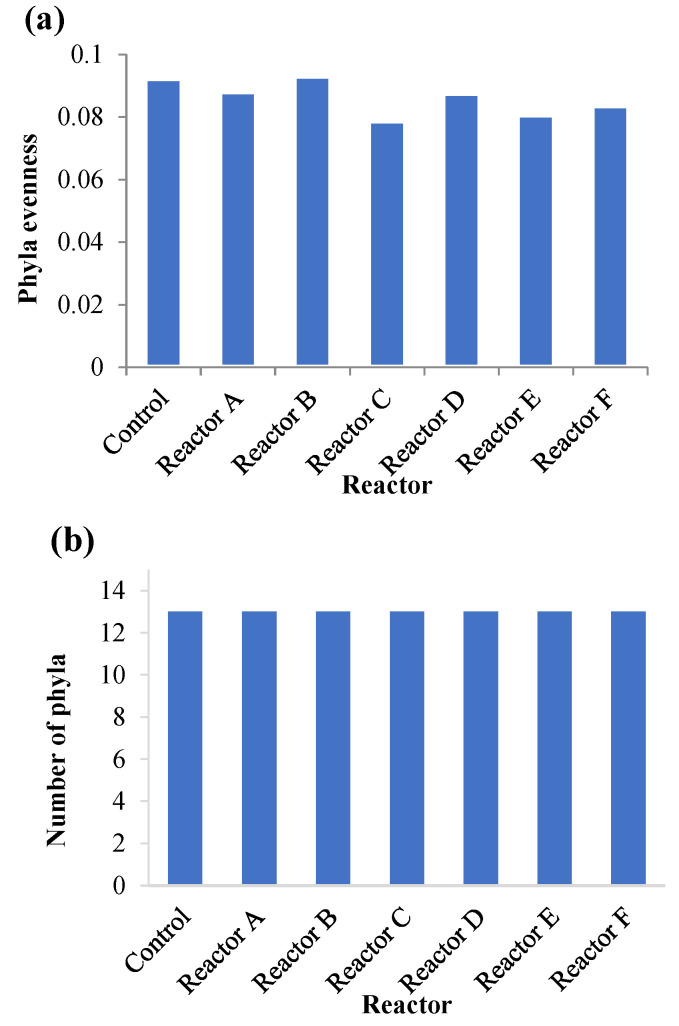
Overall phyla evenness (**a**) and phyla richness (**b**) with a relative abundance ≥ 0.01% in each reactor (reactors A, B, and C contain 2, 10, and 30 mg AgNPs/g TS, respectively, and reactors D, E, and F had 2, 10, and 30 mg CuONPs/g TS, respectively).

Phyla evenness and richness were also used to characterize the phyla population and diversity (Figure 10). The evenness is the relative abundance of phyla, and the richness represents the number of phyla in a community. As only the phyla with ≥0.01% relative abundance were considered, the phyla number was 13 in each reactor, as shown in Figure 10b. The evenness index was low and similar for all sludge reactors (CV = 4.09%). Since the evenness index determines the diversity of a bacterial community, the results in Figure 10a indicate that the reactors with AgNPs and CuONPs had slightly lower diversity compared to the control.

The bacterial community similarity was assessed through Beta-Euclidean diversity at the phyla level (Figure 8a), which was estimated by comparing the control reactor to each reactor. The difference between the control and other reactors was more pronounced when compared to the reactors with higher AgNPs or CuONPs concentrations, indicating that the presence of these nanoparticles adversely affected the bacterial diversity in the anaerobically digested sludge. Thus, all studied indices pointed to a similar outcome that high concentrations of AgNPs and CuONPs can reduce diversity at the phyla level during anaerobic digestion.

### 3.4. Abundance and Diversity of Bacteria at the Phylum Level

Three dominant phyla were identified in each reactor consisting of Proteobacteria, Firmicutes, and Bacteroidetes, as shown in Figure 8b. These results are consistent with other bacterial population studies measuring the abundance of bacterial phyla in anaerobically digested sludge [48,49,50,51]. The results showed that each of the three dominant phyla had high OTUs (between 20,000 and 50,000 reads out of ~150,000 total) with a total relative abundance of ˃98%. There were similar trends over time in all of the reactors, with no significant difference between them (*p*-value ˃ 0.05), indicative that neither AgNPs nor CuONPs seemed to significantly affect their occurrence. The next most abundant group of phyla, which comprised ~2% of the total population included Actinobacteria, Spirochaetes, Lentisphaerae, Verrucomicrobia, Planctomycetes, Chloroflexi, Fusobacteria, Fibrobacteres, Acidobacteria, and Synergistetes. When comparing the abundance of these phyla in the control reactor relative to the test reactors, slight differences in their relative abundance were observed over time, which were due to the presence of the nanoparticles; however, these differences were not statistically significant (*p*-value ˃ 0.05). Reactor B, followed by the control reactor had the highest number of observed phyla OTUs among the reactors, which may explain the lower diversity seen in test reactors with nanoparticles as shown in Figure 8c. This is in alignment with the species OTU results discussed in Section 3.3, where the presence of AgNPs and CuONPs gave rise to a higher Shannon-Wiener index and increased the overall number of phyla but lowered the observed unique OTUs at the species level within each phylum.

**Figure 11:**
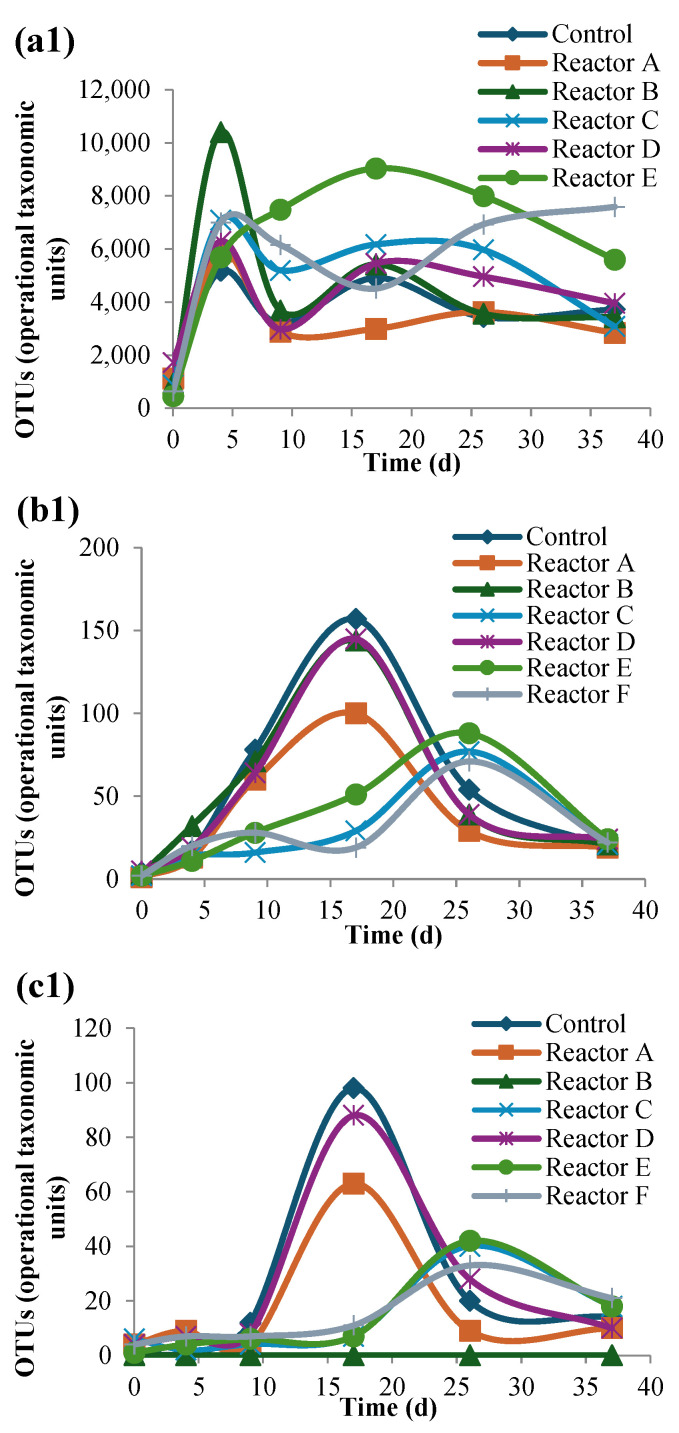

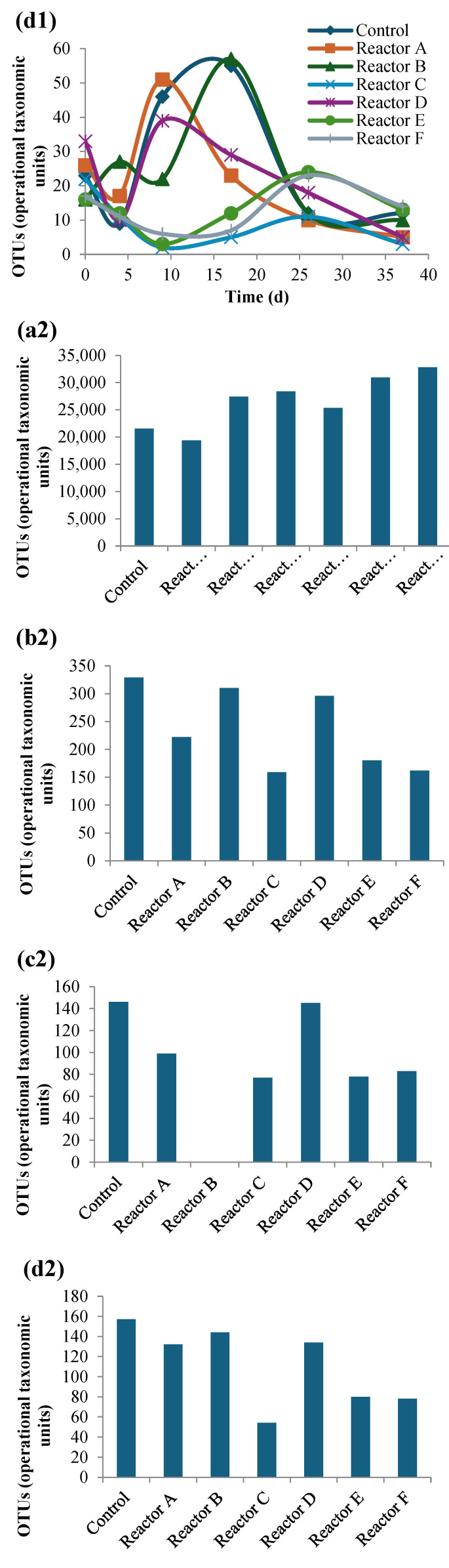
OTU over time (**1**), and total OTUs (**2**) of Bacteroidetes (**a**), Lentisphaerae (**b**), Verrucomicrobia (**c**), and Planctomycete (**d**) for each reactor (reactors A, B, and C contain 2, 10, and 30 mg AgNPs/g TS, respectively, and reactors D, E, and F contain 2, 10, and 30 mg CuONPs/g TS, respectively).

The 16S rRNA gene sequencing analysis showed that Bacteroidetes, Lentisphaerae, Verrucomicrobia, and Planctomycetes were among the phyla most impacted, all of which showed dissimilar trends over time with no significant differences between the control and test reactors (Figure 11).

### 3.5. Abundance and Diversity of Bacterial Genera

**Figure 12:**
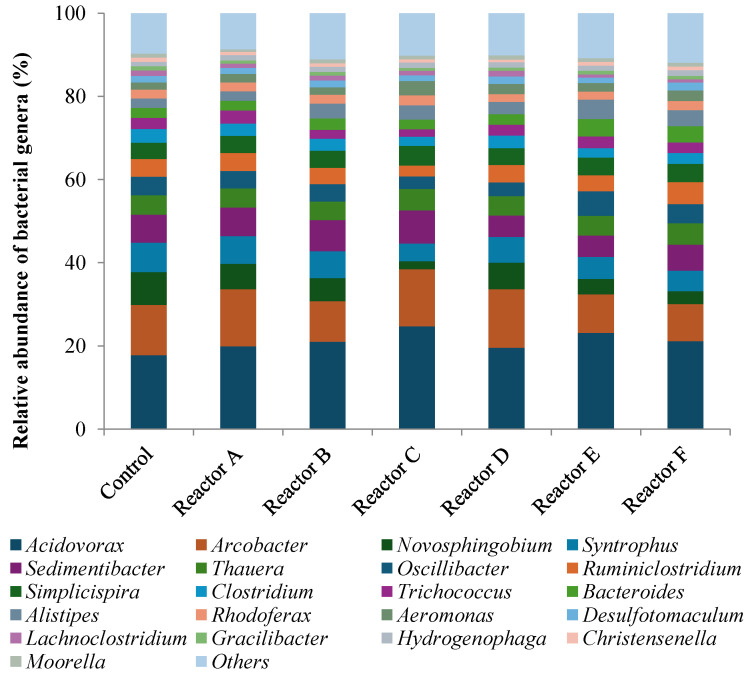
Relative abundance of the top 21 genera and those that have a relative abundance ≥1% (reactors A, B, and C contain 2, 10, and 30 mg AgNPs/g TS, respectively, and reactors D, E, and F contain 2, 10, and 30 mg CuONPs/g TS, respectively).

**Figure 13:**
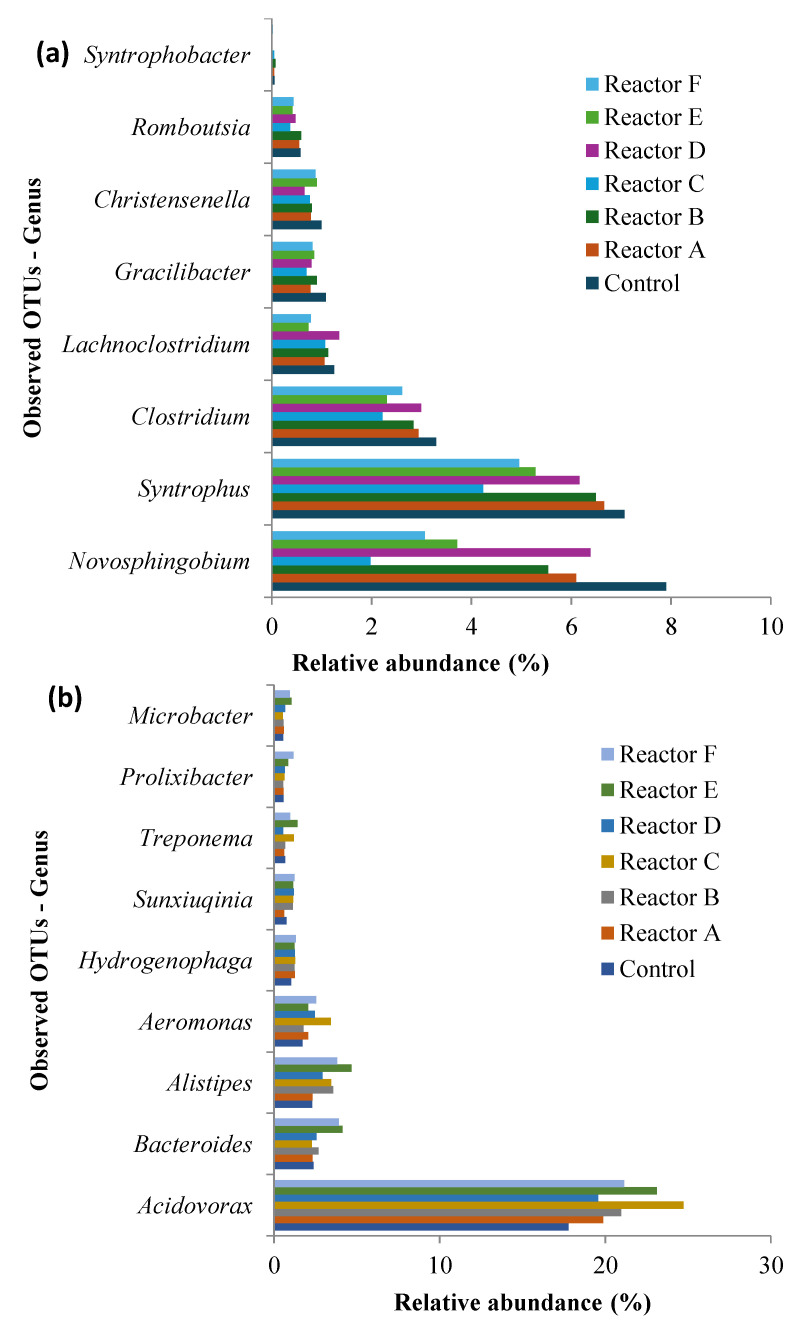
Relative abundance of 37 genera, relative abundance >0.1%, that were negatively affected by AgNPs and CuONPs (**a**), relative abundance of 37 genera, relative abundance >0.1%, that had higher observed OTUs in the presence of higher nanoparticle concentrations (**b**) (reactors A, B, and C contain 2, 10, and 30 mg AgNPs/g TS, respectively, and reactors D, E, and F contain 2, 10, and 30 mg CuONPs/g TS, respectively).

The genera discussed in this study were chosen based on their role in anaerobic digestion of sludge. The analysis showed that the total number of OTUs representing different genera detected were 504, 515, 517, 501, 461, 454, and 476 for the control and reactors A, B, C, D, E, and F, respectively. The highest concentration of AgNPs and all three concentrations of CuONPs resulted in a decreased number of genera when compared to the control, although not significantly different (*p*-value ˃ 0.05). The top 21 genera (Figure 12) represent approximately 90% of the total observed OTUs; each had ≥1% average relative abundance in all the reactors. Also, 16 genera of interest, selected from the top 75 with a relative abundance between 0.1 and 1%, were analyzed. Of the 37 (21 + 16) studied genera, several decreased in relative abundance compared to the control, in the presence of higher concentrations of AgNPs and CuONPs; these included *Novosphingobium*, *Syntrophus*, *Clostridium*, *Lachnoclostridium*, *Gracilibacter*, *Christensenella*, *Romboutsia*, and *Syntrophobacter* as shown in Figure 13a. Conversely there were genera that seemed to thrive in the reactors with higher concentrations of AgNPs and CuONPs compared to the control, which included *Acidovorax*, *Bacteroides*, *Alistipes*, *Aeromonas*, *Hydrogenophaga*, *Sunxiuqinia*, *Treponema*, *Prolixibacter*, and *Microbacter*, as shown in Figure 13b; other genera, that represented approximately 48–50% of the OTUs, did not show any change. Even though the higher concentrations of AgNPs and CuONPs affected the relative abundance of several genera as shown in Figure 13a,b, the impact was not significant, consistent with the findings of other studies [52]. However, AgNPs at a concentration of 500 mg/g TSS (very high concentration) during anaerobic digestion reduced the biogas generation by 26.5% compared to the control and inhibited the population of the bacteria and archaea assessed [25].

### 3.6. Key Bacterial Genera Involved in the Anaerobic Digestion Process

At the genus level, bacterial metagenomics can provide valuable information about the functions of communities in the sludge reactor. There are four governing stages in the anaerobic digestion process: hydrolysis, acidogenesis, acetogenesis, and methanogenesis. The efficiency and stability of the process are entirely dependent on the diversity of bacteria that are involved in each of the four stages, and their collective synergism to achieve effective sludge reduction and methane formation. Based on a literature review (Table S1, Supplementary Data), it is clear that specific bacterial genera are involved in more than one stage of the process. In this study, 37 bacterial genera selected from the top 75 genera were analyzed, and their role in anaerobic digestion was explored based on the literature, as shown in Table S1. According to the 16S rRNA gene sequencing analysis, different bacterial genera observed were found to be involved in the processes of hydrolysis, acidogenesis or acetogenesis; acidogenesis and hydrolysis; acidogenesis and acetogenesis; and hydrolysis, acidogenesis, and acetogenesis (Table 6).

**Figure 14:**
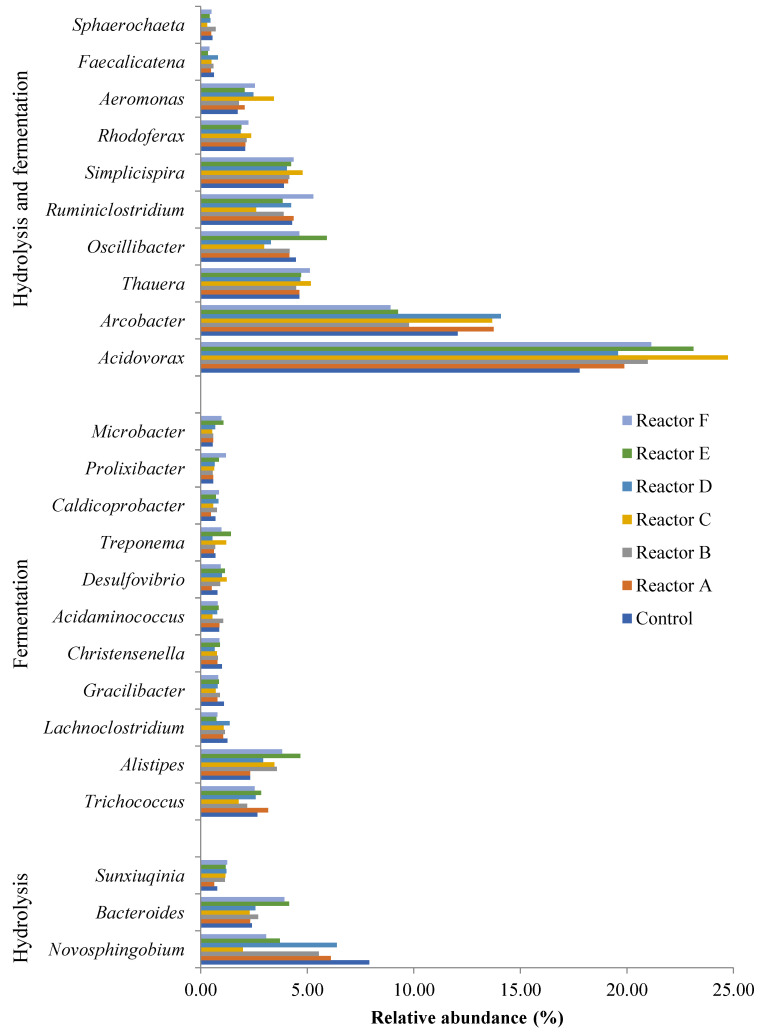
Relative abundance of bacterial genera involved in hydrolysis, acidogenesis, and hydrolysis and acidogenesis (reactors A, B, and C contain 2, 10, and 30 mg AgNPs/g TS, respectively, and reactors D, E, and F contain 2, 10, and 30 mg CuONPs/g TS, respectively).

**Figure 15:**
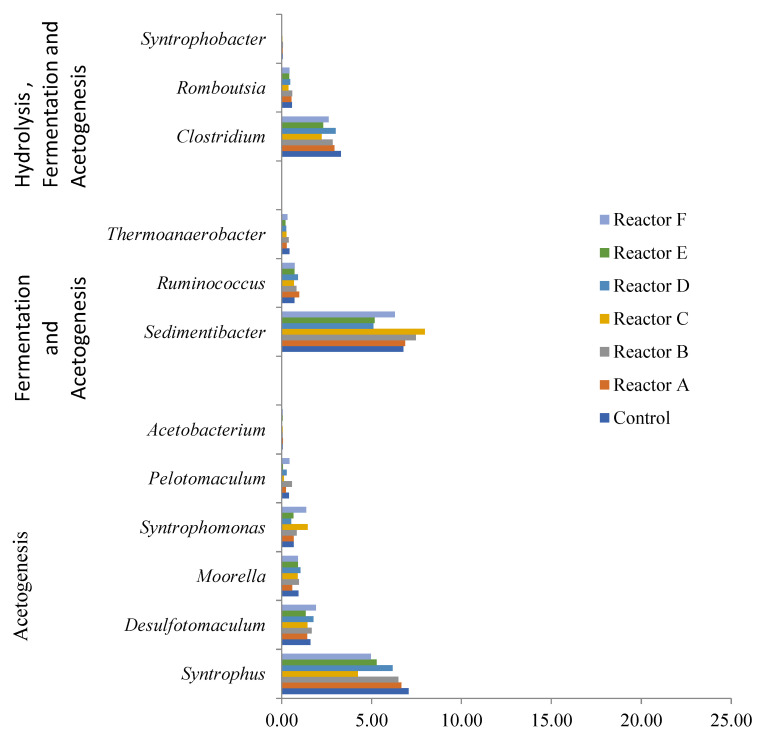
Relative abundance of bacterial genera involved in acetogenesis, acidogenesis and acetogenesis, and hydrolysis, acidogenesis, and acetogenesis (reactors A, B, and C contain 2, 10, and 30 mg AgNPs/g TS, respectively, and reactors D, E, and F contain 2, 10, and 30 mg CuONPs/g TS, respectively).

Table 6 and Figure 14 show that acidogenesis and hydrolysis had the highest relative abundance of bacterial genera in the samples. Acidogenic bacteria, mainly Proteobacteria, can assimilate amino acids, fatty acids, sugars, and alcohols, and it has been shown that this phylum is less susceptible to environmental changes than others [53], which may explain the corresponding high OTUs observed. Firmicutes phylum include a wide range of syntrophic bacteria that play an important role in anaerobic sludge digestion, including the assimilation of different volatile fatty acids (VFAs) [54]. The results also showed a high number of bacteria involved in hydrolysis and acidogenesis belonging to the Bacteroidetes phylum, which play a significant role in the anaerobic digestion process, including decomposition and conversion of organic materials into organic acids, CO_2_ and H_2_ [52,55]. The acetogenesis stage of the process was attributed to the least abundant genera assessed (0.1–1% relative abundance), which appeared to be lower in abundance in the presence of higher levels of AgNPs and CuONPs as most acetogenesis associated genera showed lower levels of observed OTUs, as shown in Figure 15.

The results also showed that the bacterial genera responsible for hydrolysis and acidogenesis (combined) had a higher relative abundance in sludge reactors containing AgNPs or CuONPs compared to the control (Table 6). For example, the genus *Acidovorax* was the highest occurring taxa in all reactors with lower OTUs in the control. In addition, the genus *Arcobacter* was found to be the second most prevalent genus in all reactors, with higher OTUs observed in some reactors that had AgNPs or CuONPs. These two genera belong to the phylum Proteobacteria and are facultative anaerobic bacteria that utilize nitrate or acetate as electron acceptors to grow under anaerobic conditions and can assimilate organic matter, acids, sugars, and proteins/amino acids [56,57,58]. The presence of AgNPs and CuONPs appeared to decrease the relative abundance of acetogenic bacterial genera, as the relative abundance of *Syntrophus*, *Sedimentibacter*, and *Clostridium* genera were lower in the reactors with higher concentrations of AgNPs or/and CuONPs. These genera represent a broad class of syntrophic bacteria that have an essential role in the anaerobic decomposition of organic matter to methane and carbon dioxide, along with fermentative and methanogenic organisms [59].

### 3.7. Metagenomic Analyses of Select Genera

**Figure 16:**
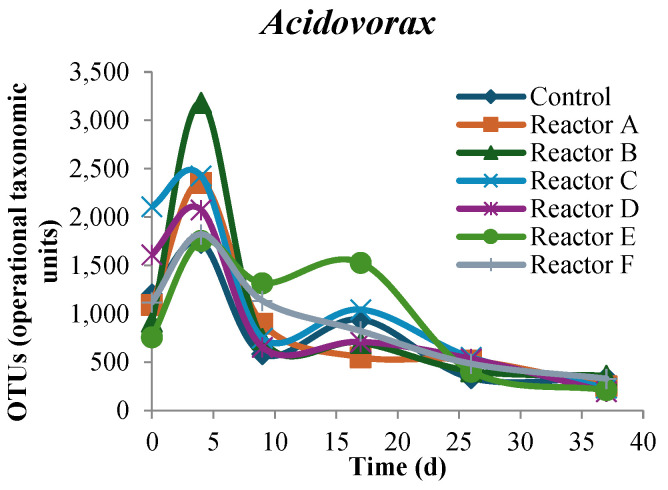

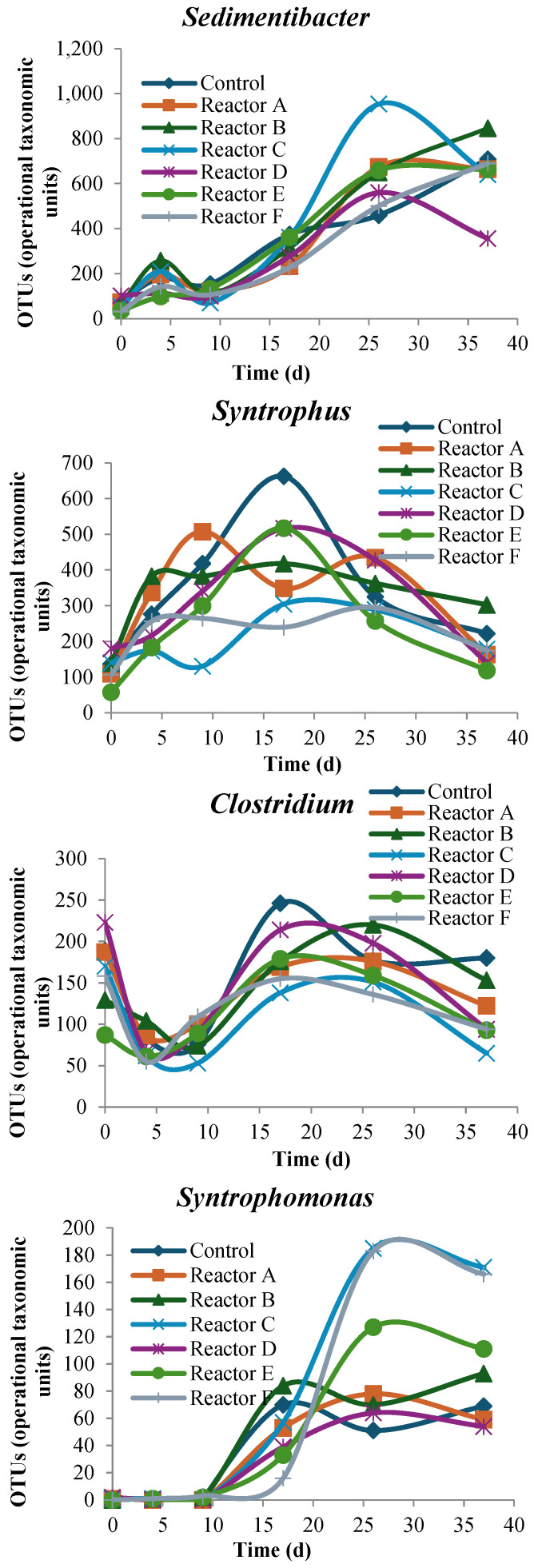

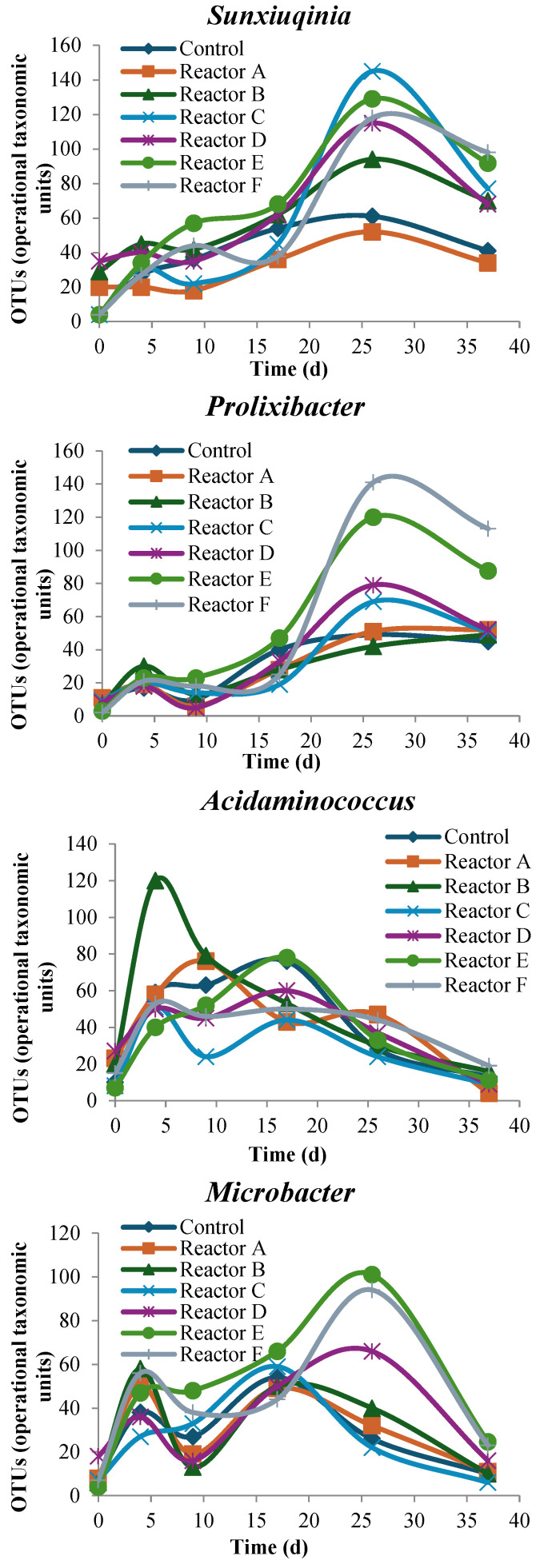

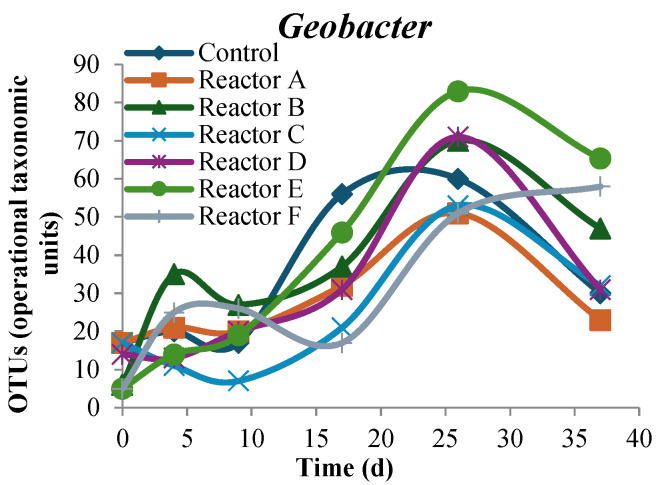
Trends of select genera over time for each reactor (reactors A, B, and C contain 2, 10, and 30 mg AgNPs/g TS, respectively, and reactors D, E, and F contain 2, 10, and 30 mg CuONPs/g TS, respectively).

Prevalence of genera OTUs over time followed similar trends overall with no significant differences among all sludge reactors. However, several genera showed different trends, which slightly varied from that of the control, including Acidovorax, Sedimentibacter, Syntrophus, Clostridium, Syntrophomonas, Sunxiuqinia, Prolixibacter, Acidaminococcus, Microbacter, and Geobacter, as shown in Figure 16. Sedimentibacter baseline OTUs were similar at the onset of the experiment but steadily increased over time, with the greatest observed OTUs in the AgNP reactors. The increase in Sedimentibacter OTU percentages on day 37 was 326, 327.2, 374.8, 381.5, 251.3, 257.6, and 283.2% for the control and reactors A, B, C, D, E, and F, respectively. Syntrophus and Clostridium showed that higher concentrations of AgNPs and CuONPs might be responsible for lower OTUs for both. For example, Syntrophus showed a 27% higher observed OTUs in reactor C compared to 62% higher in the control reactor over the 37 days, and Clostridium showed a 5% lower observed OTUs in control, while reactors C and F showed reduced observed OTUs of 62 and 40.5%, respectively. The genera Syntrophomonas and Sunxiuqinia showed higher observed OTUs over time in all reactors with higher OTU percentage prevalence in the reactors with AgNPs and CuONPs. Syntrophomonas observed OTUs increased by 17,000 and 16,600% in reactors C and F, respectively, while the control increased by 6800%. The increase in Sunxiuqinia OTUs was 1825 and 2350% for reactors C and F, respectively, compared to 925% for the control. Prolixibacter and Microbacter genera showed the highest variation in OTUs in the CuONPs reactors. The increase in Prolixibacter OTUs in reactor F was 5550% compared to 400% in the control and was 228.6% compared to 25% in the control for Microbacter. Acidaminococcus and Geobacter genera also showed similar trends over time in all reactors where higher concentrations of AgNPs and CuONPs seemed to decrease the prevalence of OTUs, as shown in Figure 16. Acidaminococcus OTUs increased by 12.5 and 27% in reactors C and F, respectively, compared to the control increase of 30%. The OTUs percentage increase in Geobacter was 88 and 1060% in reactors C and F, respectively, compared to 76.5% in control. Although there were, in some cases, marked differences in baseline OTUs over time, all sludge reactors showed similar trends after a certain amount of time. This indicates that AgNPs and CuONPs at the three tested concentrations did not have a significant impact on the population of these genera over time.

### 3.8. Discussion of the Relationship Between Bacterial Function and Digestion Performance of the Reactors

The 16S rRNA gene sequencing results (Table 6) showed that the relative abundance of the bacteria responsible for hydrolysis and acidogenesis was the highest, and increased with higher AgNP and CuONP concentrations, whereas the relative abundance of acetogenic bacteria was the least and decreased with higher nanoparticle concentrations. This is likely due to the different characteristics of bacteria that were involved in these stages, for example, hydrolytic and fermentative bacteria are phylogenetically diverse, grow rapidly, and have lower sensitivity to environmental changes, while acetogenic bacteria live in syntrophy with methanogens and are more susceptible to environmental changes [49]. Comparing the biogas generation from the reactors, the results indicated slight differences between the control and the other sludge reactors that had AgNPs and CuONPs, except the reactor that had the highest concentration of CuONPs (reactor F), which showed less generation (Figure 5). This was supported by the 16S rRNA gene sequencing analysis that showed no significant impact due to the presence of AgNPs and CuONPs. These findings agree with several studies that show the low inhibitory impact of AgNPs, while there was some inhibition due to CuONPs on methanogens [23,24]. Sulfidation of AgNPs and CuONPs under anaerobic conditions may also be an explanation for low inhibition of methanogenesis. The interaction of AgNPs and CuONPs with sulfur, and the subsequent production and precipitation of silver and copper sulfide, results in a significant reduction in their toxicity due to the lower solubility of silver and copper sulfide, which have limited short-term environmental impacts [60,61,62,63,64]. Another factor that could interfere with the antimicrobial activity of AgNPs and CuONPs is the exposure of the microbial community to high COD concentrations during the digestion process, which can diminish the antimicrobial activity and favor the growth and further acclimatization of the bacteria that were not inhibited, as well as limit the growth of some bacterial species adapted to lower COD concentrations [6]. In addition, several studies have demonstrated that AgNPs or CuONPs’ toxicity is due to the release of their ions by oxidation [65,66], which suggest that oxygen needs to be present for nanoparticles to become toxic. For example, AgNPs toxicity can be controlled by managing the availability of oxygen [66]. However, other studies have shown that the toxicity could be due to the properties of the nanoparticles themselves, including their very high surface area to volume ratio that enable them to easily bind to the bacterial cell wall and alter the membrane structure or penetrate the inside of the cell membrane and elicit further damage, including interaction with sulfur and phosphorus-containing compounds, such as proteins and DNA [67,68]. Asharani et al. [69] investigated the impact of AgNPs on red human blood cells and observed various types of cytotoxicity that were mainly due to the effect of AgNPs damaging DNA, and no toxic effects were observed when the cells were exposed to silver ions.

## 4. Conclusions

In this study, the impact of three different concentrations (2, 10, and 30 mg/g TS) of AgNPs and CuONPs on the anaerobic digestion of sludge and the bacterial community structure were investigated. Biogas generation decreased with increasing concentrations of AgNPs and CuONPs, and the decrease was greater at the highest concentration (30 mg/g TS) of both NPs. Some inhibition on the digestion of organic matter, measured by tCOD, sCOD, TS, and VS, was visible when the control reactor was compared to the reactors dosed with AgNPs and CuONPs, and particularly for the 30 mg/g TS of CuONPs reactor. CuONPs exhibited stronger inhibition of bacterial growth compared to the AgNPs evidenced by the large difference in sCOD and VS reduction. After the addition of AgNPs and CuONPs, cell viability gradually decreased over the first two weeks but recovered after that. The decreases in cell viability were noticeable with increasing concentrations of NPs, particularly for CuONPs, but were not statistically significant. At high concentrations, AgNPs and CuONPs decreased the overall species diversity, and inhibited the dominant bacterial species, allowing the less abundant species to increase. The relative abundance of the bacteria responsible for hydrolysis and acidogenesis increased and the relative abundance of acetogenic bacteria decreased with higher AgNP and CuONP concentrations. Compared to the AgNPs, CuONPs had a stronger adverse effect on the bacterial community structure, diversity, abundance, and functions. The majority of the parameters measured for monitoring the anaerobic digestion performance and bacterial community were not statistically significant at the lowest 2 mg/g TS of AgNPs and CuONPs concentrations tested in this study, which represents naturally present concentrations in wastewater sludge.

## Figures and Tables

**Table 1:** AgNPs and CuONPs characteristics.

Characteristics	AgNPs	CuONPs
Diameter (TEM)	23.1 ± 6.9 nm	25–55 nm
pH of the solution	6.3	7–9
Morphology	Nearly spherical	Nearly spherical
Solvent	Milli-Q water (Merck, Darmstadt, Germany)	Milli-Q water
Particle surface coating	Polyvinylpyrrolidone (PVP)	None

**Table 2:** Characteristics of primary, tWAS, and anaerobically digested sludge.

Parameters			
	Primary Sludge	Thickened Waste Activated Sludge (tWAS)	Anaerobically Digested Sludge
pH	7.6 ± 0.1	7.0 ± 0.1	7.3 ± 0.02
Total solids (TS) (g/kg)	42.7 ± 7.2	52.7 ± 1.6	19.0 ± 0.2
VS (g/kg)	28.7 ± 3.5	38.6 ± 1.5	15.6 ± 0.1
Total chemical oxygen demand (tCOD) (g/kg)	52.0 ± 8.3	73.9 ± 2.1	19.8 ± 1.1
Soluble chemical oxygen demand (sCOD) (g/kg)	2.0 ± 1.1	8.2 ± 0.1	8.4 ± 0.2

Values show the mean of three replicate measurements ± standard deviation.

**Table 3:** Experimental conditions for the BMP reactors.

Reactors	NP Concentration (mg NPs/g TS of Sludge)	Sludge Feed	Reactor Volume (mL)	Reactor Temperature (°C)	Duration of Experiments (days)
Control	Zero	Thickened waste activated sludge (40%) + primary sludge (40%) + thickened anaerobically digested sludge (20%)	85	34 ± 1	37
Reactor A (AgNPs)	2				
Reactor B (AgNPs)	10				
Reactor C (AgNPs)	30				
Reactor D (CuONPs)	2				
Reactor E (CuONPs)	10				
Reactor F (CuONPs)	30				

**Table 4:** Biogas volume, tCOD, sCOD and VS reduction in the BMP reactors.

Reactor	Final Biogas Volume Generated (mL)	tCOD Reduction %	sCOD Reduction %	VS Reduction %
Control	800.5 ± 3	56.4 ± 4.2	71.1 ± 5.2	50.0 ± 3.8
Reactor A (2 mg AgNPs/g TS)	704.0 ± 4	50.8 ± 2.7	70.3 ± 7.1	50.1 ± 1.3
Reactor B (10 mg AgNPs/g TS)	689.0 ± 5	43.9 ± 2.2	70.0 ± 5.5	46.2 ± 2.1
Reactor C (30 mg AgNPs/g TS)	580.5 ± 3	51.3 ± 1.7	70.0 ± 3.4	49.8 ± 1.8
Reactor D (2 mg CuONPs/g TS)	769.0 ± 2	53.1 ± 2.1	59.0 ± 6.4	43.6 ± 2.4
Reactor E (10 mg CuONPs/g TS)	768.0 ± 3	52.1 ± 3.1	59.5 ± 7.1	41.2 ± 4.1
Reactor F (30 mg CuONPs/g TS)	562.5 ± 2	50.8 ± 3.2	61.9 ± 6.1	41.9 ± 3.4

Data represents the mean ± standard deviation of replicate measurements.

**Table 5:** Overall diversity of the microbial population in the BMP reactors (all time points combined).

Reactor	Nanoparticles Concentration (mg NPs/g TS of Sludge)	Overall Number of Sequences	Classified (%)	Total Number of Observed Phyla	Average Process Accuracy (%)	Shannon-Wiener Index (Phyla)
Control	None	148,735	83	24	83	1.15
Reactor A (AgNPs)	2	147,632	76	27	82	1.12
Reactor B (AgNPs)	10	149,972	87	27	83	1.16
Reactor C (AgNPs)	30	143,490	87	27	83	1.14
Reactor D (CuONPs)	2	141,444	89	27	83	1.14
Reactor E (CuONPs)	10	136,469	86	29	83	1.17
Reactor F (CuONPs)	30	148,759	85	23	83	1.17

**Table 6:** Relative abundance of bacterial genera involved in different stages of the anaerobic.

Bacterial Phylum/Genera	Control (%)	Reactor A (%)	Reactor B (%)	Reactor C (%)	Reactor D (%)	Reactor E (%)	Reactor F (%)
Proteobacteria/*Novosphingobium*. Bacteroidetes/*Bacteroides* and *Sunxiuqinia*.	11.1	9.1	9.4	5.4	10.2	9.0	8.2
Firmicutes/*Trichococcus*, *Lachnoclostridium*, *Gracilibacter*, *Christensenella*, *Acidaminococcus*, *Desulfovibrio*, *Treponema*, and *Caldicoprobacter*. Bacteroidetes/*Alistipes*, *Prolixibacter*, and *Microbacter*	12.5	11.8	13.2	12.5	12.8	16.0	14.6
Proteobacteria/*Syntrophus*. Firmicutes/*Desulfotomaculum*, *Moorella*, *Syntrophomonas*, *Pelotomaculum*, and *Acetobacterium*.	10.7	9.6	10.6	8.2	9.8	8.3	9.6
Proteobacteria/*Acidovorax*, *Arcobacter*, *Thauera*, *Simplicispira*, *Rhodoferax*, and *Aeromonas*. Firmicutes/*Oscillibacter*, *Ruminiclostridium*, and *Faecalicatena*. Spirochaetes/*Sphaerochaeta*.	52.1	56.0	52.7	60.5	55.5	55.8	55.2
Firmicutes/*Sedimentibacter*, *Ruminococcus*, and *Thermoanaerobacter*.	7.9	8.1	8.7	8.9	6.3	6.1	7.3
Firmicutes/*Clostridium*, *Romboutsia*, and *Syntrophobacter*.	3.9	3.5	3.5	2.6	3.5	2.7	3.1

## Data Availability

Data are contained within the article or Supplementary Materials.

## Supplementary Materials

The following supporting information can be downloaded at: https://www.mdpi.com/article/10.3390/nano15030236/s1, Figure S1: Live/Dead cells images for the control over time; Figure S2: Live/Dead cells images for reactor A (2 mg AgNPs/g TS of sludge); Figure S3: Live/Dead cells images for reactor B (10 mg AgNPs/g TS of sludge); Figure S4: Live/Dead cells images for reactor C (30 mg AgNPs/g TS of sludge); Figure S5: Live/Dead cells images for reactor D (2 mg AgNPs/g TS of sludge); Figure S6: Live/Dead cells images for reactor E (10 mg AgNPs/g TS of sludge); Figure S7: Live/Dead cells images for reactor F (30 mg AgNPs/g TS of sludge); Table S1: Genus of interest and their role in the sludge anaerobic digestion process. References [70,71,72,73,74,75,76,77,78,79,80,81,82,83,84,85,86,87,88,89,90,91,92,93,94,95,96,97,98,99,100,101,102,103,104,105,106] are cited in the supplementary materials.

## References

[1] Battez A.V., González R., Blanco D., Asedegbega E., Osorio A. (2010). Friction reduction properties of a CuO nanolubricant used as lubricant for a NiCrBSi coating. Wear.

[2] Babu S., Claville M.O., Ghebreyessus K. (2015). Rapid synthesis of highly stable silver nanoparticles and its application for colourimetric sensing of cysteine. J. Exp. Nanosci..

[3] (2017). US Research Nanomaterials Inc.. https://www.us-nano.com/inc/sdetail/602.

[4] Vasilie G., Kubo A.-L., Vija H., Kahru A., Bondar D., Karpichev Y., Bondarenko O. (2023). Synergistic antibacterial effect of copper and silver nanoparticles and their mechanism of action. Sci. Rep..

[5] Jo H.J., Choi J.W., Lee S.H., Hong S.W. (2012). Acute toxicity of Ag and CuO nanoparticle suspensions against Daphnia magna: The importance of their dissolved fraction varying with preparation methods. J. Hazard. Mater..

[6] Jang S.-W., Oh M.-S., Yang S.I., Cho E.-M. (2016). Gene expression profiles of human neuroblastoma cells exposed to CuO nanoparticles and Cu ions. BioChip J..

[7] Juling S., Niedzwiecka A., Böhmert L., Lichtenstein D., Selve S., Braeuning A., Thünemann A.F., Krause E., Lampen A. (2017). Protein corona analysis of silver nanoparticles links to their cellular effects. J. Proteome Res..

[8] Yilmaz G.E., Gokturk I., Ovezova M., Yilmaz F., Kilic S., Denizli A. (2023). Antimicrobial Nanomaterials: A Review. Hygiene.

[9] Lee Y.J., Lee D.J. (2019). Impact of adding metal nanoparticles on anaerobic digestion performance—A review. Bioresour. Technol..

[10] Gottschalk F., Tobias S., Roland W.S., Bernd N. (2009). Modeled environmental concentrations of engineered nanomaterials (TiO2, ZnO, Ag, CNT, fullerenes) for different regions. Environ. Sci. Technol..

[11] Keller A.A., McFerran S., Lazareva A., Suh S. (2013). Global life cycle releases of engineered nanomaterials. J. Nanoparticle Res..

[12] Shafer M.M., Overdier J.T., Armstong D.E. (1998). Removal, partitioning, and fate of silver and other metals in wastewater treatment plants and effluent-receiving streams. Environ. Toxicol. Chem..

[13] Kaegi R., Voegelin A., Sinnet B., Zuleeg S., Hagendorfer H., Burkhardt M., Siegrist H. (2011). Behavior of metallic silver nanoparticles in a pilot wastewater treatment plant. Environ. Sci. Technol..

[14] Li L., Stoiber M., Wimmer A., Xu Z., Lindenblatt C., Helmreich B., Schuster M. (2016). To what extent can full-scale wastewater treatment plant effluent influence the occurrence of silver based nanoparticles in surface waters?. Environ. Sci. Technol..

[15] Epelle E.I., Ukoye P.U., Roddy S., Gunes B., Okolie J.A. (2022). Advances in the Applications of Nanomaterials for Wastewater Treatment. Environments.

[16] Liang Z., Das A., Hu Z. (2010). Bacterial response to a shock load of nanosilver in an activated sludge treatment system. Water Res..

[17] Sun X., Sheng Z., Liu Y. (2013). Effects of silver nanoparticles on microbial community structure in activated sludge. Sci. Total Environ..

[18] Qiu G., Wirianto K., Sun Y., Ting Y.-P. (2016). Effect of silver nanoparticles on system performance and microbial community dynamics in a sequencing batch reactor. J. Clean. Prod..

[19] Zhang D., Trzcinski A.P., Oh H.-S., Chew E., Tan S.K., Ng W.J., Liu Y. (2017). Comparison and distribution of copper oxide nanoparticles and copper ions in activated sludge reactors. J. Environ. Sci. Health Part A.

[20] Luna-delRisco M., Orupõld K., Dubourguier H.-C. (2011). Particle-size effect of CuO and ZnO on biogas and methane production during anaerobic digestion. J. Hazard. Mater..

[21] Wang D., Chen Y. (2016). Critical review of the influences of nanoparticles on biological wastewater treatment and sludge digestion. Crit. Rev. Biotechnol..

[22] Rasool K., Lee D.S. (2016). Inhibitory effects of silver nanoparticles on removal of organic pollutants and sulfate in an anaerobic biological wastewater treatment process. J. Nanosci. Nanotechnol..

[23] Gonzalez-Estrella J., Sierra-Alvarez R., Field J.A. (2013). Toxicity assessment of inorganic nanoparticles to acetoclastic and hydrogenotrophic methanogenic activity in anaerobic granular sludge. J. Hazard. Mater..

[24] Ünşar E., Çığgın A., Erdem A., Perendeci N. (2016). Long and short term impacts of CuO, Ag and CeO2 nanoparticles on anaerobic digestion of municipal waste activated sludge. Environ. Sci. Process. Impacts.

[25] Wang T., Zhang D., Dai L., Chen Y., Dai X. (2016). Effects of Metal Nanoparticles on Methane Production from Waste-Activated Sludge and Microorganism Community Shift in Anaerobic Granular Sludge. Sci. Rep..

[26] Jeyakumar R.B., Vincent G.S. (2022). Recent Advances and Perspectives of Nanotechnology in Anaerobic Digestion: A New Paradigm towards Sludge Biodegradability. Sustainability.

[27] Blaser S.A., Martin S., Matthew M., Konrad H. (2008). Estimation of cumulative aquatic exposure and risk due to silver: Contribution of nano-functionalized plastics and textiles. Sci. Total Environ..

[28] US EPA (2009). Targeted National Sewage Sludge Survey Sampling and Analysis Technical Report.

[29] CCME (2020). Review of the Current Canadian Legislative Framework for Wastewater Biosolids.

[30] Gottschalk F., Sun T., Nowack B. (2013). Environmental concentrations of engineered nanomaterials: Review of modeling and analytical studies. Environ. Pollut..

[31] Rice E., Baird R., Eaton A., APHA, AWWA, WEF (2017). Standard Methods for the Examination of Water and Wastewater.

[32] Kibbee R.J., Örmeci B. (2017). Development of a sensitive and false-positive free PMA-qPCR viability assay to quantify VBNC Escherichia coli and evaluate disinfection performance in wastewater effluent. J. Microbiol. Methods.

[33] Magurran A.E. (1988). Ecological Diversity and Its Measurement.

[34] Mulder C.P., Bazeley-White E., Dimitrakopoulos P.G., Hector A., Scherer-Lorenzen M., Schmid B. (2004). Species evenness and productivity in experimental plant communities. Oikos.

[35] Edgar R. Usearch V11, Euclidean Distance Metric. 2018. Retrieved from drive5 Bioinformatics Software and Services. https://drive5.com/usearch/manual/euclidean_distance.html.

[36] Currell G., Dowman A. (2005). Essential Mathematics and Statistics for Science, Chichester.

[37] Hou L., Li K., Ding Y., Li Y., Chen J., Wu X., Li X. (2012). Removal of silver nanoparticles in simulated wastewater treatment processes and its impact on COD and NH4 reduction. Chemosphere.

[38] Zhang L.H., Zeng G.M., Dong H.R., Chen Y.N., Zhang J.C., Yan M., Zhu Y., Yuan Y.J., Xie Y.K., Huang Z.Z. (2017). The impact of silver nanoparticles on the co-composting of sewage and agricultural waste: Evolutions of organic matter and nitrogen. Bioresour. Technol..

[39] Yang Y., Chen Q., Wall J., Hu Z. (2012). Potential nanosilver impact on anaerobic digestion at moderate silver concentrations. Water Res..

[40] Sakarya K., Akyol Ç., Demirel B. (2015). The effect of short-term exposure of engineered nanoparticles on methane production during mesophilic anaerobic digestion of primary sludge. Water Air Soil Pollut..

[41] Otero-González L., Field J.A., Sierra-Alvarez R. (2014). Inhibition of anaerobic wastewater treatment after long-term exposure to low levels of CuO nanoparticles. Water Res..

[42] Kaweeteerawat C., Ubol P.N., Sangmuang S., Aueviriyavit S., Maniratanachote R. (2017). Mechanisms of antibiotic resistance in bacteria mediated by silver nanoparticles. J. Toxicol. Environ. Health Part A.

[43] Hachicho N., Hoffmann P., Ahlert K., Heipieper H.J. (2014). Effect of silver nanoparticles and silver ions on growth and adaptive response mechanisms of Pseudomonas putida mt-2. FEMS Microbiol. Lett..

[44] Huang K., Tang J., Zhang X.-X., Xu K., Ren H. (2014). A comprehensive insight into tetracycline resistant bacteria and antibiotic resistance genes in activated sludge using next-generation sequencing. Int. J. Mol. Sci..

[45] Li D., Qi R., Yang M., Zhang Y., Yu T. (2011). Bacterial community characteristics under long-term antibiotic selection pressures. Water Res..

[46] Czárán T.L., Hoekstra R.F., Pagie L. (2002). Chemical warfare between microbes promotes biodiversity. Proc. Natl. Acad. Sci. USA.

[47] Huang H., Chen Y., Yang S., Zheng X. (2019). CuO and ZnO nanoparticles drive the propagation of antibiotic resistance genes during sludge anaerobic digestion: Possible role of stimulated signal transduction. Environ. Sci..

[48] Kirkegaard R.H., McIlroy S.J., Kristensen J.M., Nierychlo M., Karst S.M., Dueholm M.S., Albertsen M., Nielsen P.H. (2017). Identifying the abundant and active microorganisms common to full scale anaerobic digesters. bioRxiv.

[49] Venkiteshwaran K., Bocher B., Maki J., Zitomer D. (2015). Relating anaerobic digestion microbial community and process function: Supplementary issue: Water microbiology. Microbiol. Insights.

[50] Guo J., Peng Y., Ni B.-J., Han X., Fan L., Yuan Z. (2015). Dissecting microbial community structure and methane-producing pathways of a full-scale anaerobic reactor digesting activated sludge from wastewater treatment by metagenomic sequencing. Microb. Cell Factories.

[51] Yang Y., Quensen J., Mathieu J., Wang Q., Wang J., Li M., Tiedje J.M., Alvarez P.J. (2014). Pyrosequencing reveals higher impact of silver nanoparticles than Ag+ on the microbial community structure of activated sludge. Water Res..

[52] Doolette C.L., McLaughlin M.J., Kirby J.K., Batstone D.J., Harris H.H., Ge H., Cornelis G. (2013). Transformation of PVP coated silver nanoparticles in a simulated wastewater treatment process and the effect on microbial communities. Chem. Cent. J..

[53] Ariesyady H.D., Ito T., Okabe S. (2007). Functional bacterial and archaeal community structures of major trophic groups in a full-scale anaerobic sludge digester. Water Res..

[54] Garcia-Peña E.I., Parameswaran P., Kang D.W., Canul-Chan M., Krajmalnik-Brown R. (2011). Anaerobic digestion and co-digestion processes of vegetable and fruit residues: Process and microbial ecology. Bioresour. Technol..

[55] Traversi D., Villa S., Lorenzi E., Degan R., Gilli G. (2012). Application of a real-time qPCR method to measure the methanogen concentration during anaerobic digestion as an indicator of biogas production capacity. J. Environ. Manag..

[56] Schulze R., Spring S., Amann R., Huber I., Ludwig W., Schleifer K.-H., Kämpfer P. (1999). Genotypic diversity of Acidovorax strains isolated from activated sludge and description of *Acidovorax defluvii* sp, nov. Syst. Appl. Microbiol..

[57] Vandamme P., Vancanneyt M., Pot B., Mels L., Hoste B., Dewettinck D., Vlaes L., Borre C.V.D., Higgins R., Hommez J. (1992). Polyphasic taxonomic study of the emended genus Arcobacter with *Arcobacter butzleri* comb. nov. and *Arcobacter skirrowii* sp. nov., an aerotolerant bacterium isolated from veterinary specimens. Int. J. Syst. Evol. Microbiol..

[58] Roalkvam I., Drønen K., Stokke R., Daae F.L., Dahle H., Steen I.H. (2015). Physiological and genomic characterization of Arcobacter anaerophilus IR-1 reveals new metabolic features in Epsilonproteobacteria. Front. Microbiol..

[59] McInerney M.J., Rohlin L., Mouttaki H., Kim U., Krupp R.S., Rios-Hernandez L., Sieber J., Struchtemeyer C.G., Bhattacharyya A., Campbell J.W. (2007). The genome of Syntrophus aciditrophicus: Life at the thermodynamic limit of microbial growth. Proc. Natl. Acad. Sci. USA.

[60] Kim B., Park C.-S., Murayama M., Hochella M.F. (2010). Discovery and characterization of silver sulfide nanoparticles in final sewage sludge products. Environ. Sci. Technol..

[61] Levard C., Hotze E.M., Lowry G.V., Brown G.E. (2012). Environmental transformations of silver nanoparticles: Impact on stability and toxicity. Environ. Sci. Technol..

[62] Kaegi R., Voegelin A., Ort C., Sinnet B., Thalmann B., Krismer J., Hagendorfer H., Elumelu M., Mueller E. (2013). Fate and transformation of silver nanoparticles in urban wastewater systems. Water Res..

[63] Lombi E., Donner E., Taheri S., Tavakkoli E., Jämting Å.K., McClure S., Naidu R., Miller B.W., Scheckel K.G., Vasilev K. (2013). Transformation of four silver/silver chloride nanoparticles during anaerobic treatment of wastewater and post-processing of sewage sludge. Environ. Pollut..

[64] Gonzalez-Estrella J., Puyol D., Sierra-Alvarez R., Field J.A. (2015). Role of biogenic sulfide in attenuating zinc oxide and copper nanoparticle toxicity to acetoclastic methanogenesis. J. Hazard. Mater..

[65] Bondarenko O., Ivask A., Käkinen A., Kahru A. (2012). Sub-toxic effects of CuO nanoparticles on bacteria: Kinetics, role of Cu ions and possible mechanisms of action. Environ. Pollut..

[66] Xiu Z.-M., Zhang Q.-B., Puppala H.L., Colvin V.L., Alvarez P.J. (2012). Negligible particle-specific antibacterial activity of silver nanoparticles. Nano Lett..

[67] Klasen H.J. (2000). A historical review of the use of silver in the treatment of burns, II. Renewed interest for silver. Burns.

[68] Lansdown A.B. (2004). A review of the use of silver in wound care: Facts and fallacies. Br. J. Nurs..

[69] Asharani P.V., Sethu S., Vadukumpully S., Zhong S., Lim C.T., Hande M.P., Valiyaveettil S. (2010). Investigations on the structural damage in human erythrocytes exposed to silver, gold, and platinum nanoparticles. Adv. Funct. Mater..

[70] Ginige M.P., Keller J., Blackall L.L. (2005). Investigation of an acetate-fed denitrifying microbial community by stable isotope probing, full-cycle rRNA analysis, and fluorescent in situ hybridization-microautoradiography. Appl. Environ. Microbiol..

[71] Takeuchi M., Hamana K., Hiraishi A. (2001). Proposal of the genus Sphingomonas sensu stricto and three new genera, Sphingobium, Novosphingobium and Sphingopyxis, on the basis of phylogenetic and chemotaxonomic analyses. Int. J. Syst. Evol. Microbiol..

[72] Elshahed M.S., McInerney M.J. (2001). Benzoate Fermentation by the Anaerobic Bacterium *Syntrophus aciditrophicus* in the Absence of Hydrogen-Using Microorganisms. Appl. Environ. Microbiol..

[73] Breitenstein A., Wiegel J., Haertig C., Weiss N., Andreesen J.R., Lechner U. (2002). Reclassification of Clostridium hydroxybenzoicum as *Sedimentibacter hydroxybenzoicus* gen. nov., comb. nov., and description of *Sedimentibacter saalensis* sp. nov. Int. J. Syst. Evol. Microbiol..

[74] Garrity G., Brenner D.K., Staley J., Krieg N. (2005). The Proteobacteria, Part C: The Alpha-, Beta-, Delta-, and Epsilonproteobacteria. Bergey’s Manual of Systematic Bacteriology.

[75] Iino T., Mori K., Tanaka K., Suzuki K.-i., Harayama S. (2007). *Oscillibacter valericigenes* gen. nov., sp. nov., a valerate-producing anaerobic bacterium isolated from the alimentary canal of a Japanese corbicula clam. Int. J. Syst. Evol. Microbiol..

[76] Fosses A., Maté M., Franche N., Liu N., Denis Y., Borne R., de Philip P., Fierobe H.-P., Perret S. (2017). A seven-gene cluster in *Ruminiclostridium cellulolyticum* is essential for signalization, uptake and catabolism of the degradation products of cellulose hydrolysis. Biotechnol. Biofuels.

[77] Grabovich M., Gavrish E., Kuever J., Lysenko A.M., Podkopaeva D., Dubinina G. (2006). Proposal of *Giesbergeria voronezhensis* gen. nov., sp. nov. and *G. kuznetsovii* sp. nov. and reclassification of *[Aquaspirillum] anulus*, *[A.] sinuosum* and *[A.] giesbergeri* as *Giesbergeria anulus* comb. nov., *G. sinuosa* comb. nov. and *G. giesbergeri* comb. nov. Int. J. Syst. Evol. Microbiol..

[78] Wiegel J., Tanner R., Rainey F. (2006). An Introduction to the Family Clostridiaceae.

[79] Pikuta E.V., Hoover R.B., Bej A.K., Marsic D., Whitman W.B., Krader P.E., Tang J. (2006). *Trichococcus patagoniensis* sp. nov., a facultative anaerobe that grows at −5 °C, isolated from penguin guano in Chilean Patagonia. Int. J. Syst. Evol. Microbiol..

[80] Li A., Chu Y., Wang X., Ren L., Yu J., Liu X., Yan J., Zhang L., Wu S., Li S. (2013). A pyrosequencing-based metagenomic study of methane-producing microbial community in solid-state biogas reactor. Biotechnol. Biofuels.

[81] Nagai F., Morotomi M., Watanabe Y., Sakon H., Tanaka R. (2010). *Alistipes indistinctus* sp. nov. and *Odoribacter laneus* sp. nov., common members of the human intestinal microbiota isolated from faeces. Int. J. Syst. Evol. Microbiol..

[82] Finneran K.T., Johnsen C.V., Lovley D.R. (2003). *Rhodoferax ferrireducens* sp. nov., a psychrotolerant, facultatively anaerobic bacterium that oxidizes acetate with the reduction of Fe (III). Int. J. Syst. Evol. Microbiol..

[83] Abbott S.L., Cheung W.K., Janda J.M. (2003). The genus Aeromonas: Biochemical characteristics, atypical reactions, and phenotypic identification schemes. J. Clin. Microbiol..

[84] Aullo T., Ranchou-Peyruse A., Ollivier B., Magot M. (2013). *Desulfotomaculum* spp. and related gram-positive sulfate-reducing bacteria in deep subsurface environments. Front. Microbiol..

[85] Yutin N., Galperin M.Y. (2013). A genomic update on clostridial phylogeny: Gram-negative spore formers and other misplaced clostridia. Environ. Microbiol..

[86] Chung B.S., Ryu S.H., Park M., Jeon Y., Chung Y.R., Jeon C.O. (2007). *Hydrogenophaga caeni* sp. nov., isolated from activated sludge. Int. J. Syst. Evol. Microbiol..

[87] Lee Y.-J., Romanek C.S., Mills G.L., Davis R.C., Whitman W.B., Wiegel J. (2006). *Gracilibacter thermotolerans* gen. nov., sp. nov., an anaerobic, thermotolerant bacterium from a constructed wetland receiving acid sulfate water. Int. J. Syst. Evol. Microbiol..

[88] Morotomi M., Nagai F., Watanabe Y. (2012). Description of *Christensenella minuta* gen. nov., sp. nov., isolated from human faeces, which forms a distinct branch in the order Clostridiales, and proposal of *Christensenellaceae* fam. nov. Int. J. Syst. Evol. Microbiol..

[89] Islam M.A., Zengler K., Edwards E.A., Mahadevan R., Stephanopoulos G. (2015). Investigating *Moorella thermoacetica* metabolism with a genome-scale constraint-based metabolic model. Integr. Biol..

[90] Euzéby J.P. (1997). List of Bacterial Names with Standing in Nomenclature: A folder available on the Internet. J. Syst. Evol. Microbiol..

[91] Takai K., Abe M., Miyazaki M., Koide O., Nunoura T., Imachi H., Inagaki F., Kobayashi T. (2013). *Sunxiuqinia faeciviva* sp. nov., a facultatively anaerobic organoheterotroph of the Bacteroidetes isolated from deep subseafloor sediment. Int. J. Syst. Evol. Microbiol..

[92] Thabet O.B., Fardeau M.-L., Suarez-Nuñez C., Hamdi M., Thomas P., Ollivier B., Alazard D. (2007). *Desulfovibrio marinus* sp. nov., a moderately halophilic sulfate-reducing bacterium isolated from marine sediments in Tunisia. Int. J. Syst. Evol. Microbiol..

[93] Hatamoto M., Imachi H., Fukayo S., Ohashi A., Harada H. (2007). *Syntrophomonas palmitatica* sp. nov., an anaerobic, syntrophic, long-chain fatty-acid-oxidizing bacterium isolated from methanogenic sludge. Int. J. Syst. Evol. Microbiol..

[94] Ben David Y., Dassa B., Borovok I., Lamed R., Koropatkin N.M., Martens E.C., White B.A. (2015). Ruminococcal cellulosome systems from rumen to human. Environ. Microbiol..

[95] Veldkamp H. (1960). Isolation and characteristics of *Treponema zuelzerae* nov. spec., an anaerobic, free-living spirochete. Antonie Van Leeuwenhoek.

[96] Bouanane-Darenfed A., Hania W.B., Cayol J.-L., Ollivier B., Fardeau M.-L. (2015). Reclassification of Acetomicrobium faecale as *Caldicoprobacter faecalis* comb. nov. Int. J. Syst. Evol. Microbiol..

[97] Sakamoto M., Iino T., Ohkuma M. (2017). *Faecalimonas umbilicata* gen. nov., sp. nov., isolated from human faeces, and reclassification of *Eubacterium contortum*, *Eubacterium fissicatena* and *Clostridium oroticum* as *Faecalicatena contorta* gen. nov., comb. nov., *Faecalicatena fissicatena* comb. nov. Int. J. Syst. Evol. Microbiol..

[98] Caccavo F., Lonergan D.J., Lovley D.R., Davis M., Stolz J.F., McInerney M.J. (1994). Geobacter sulfurreducens sp. nov., a hydrogen-and acetate-oxidizing dissimilatory metal-reducing microorganism. Appl. Environ. Microbiol..

[99] Holmes D.E., Nevin K.P., Woodard T.L., Peacock A.D., Lovley D.R. (2007). *Prolixibacter bellariivorans* gen. nov., sp. nov., a sugar-fermenting, psychrotolerant anaerobe of the phylum Bacteroidetes, isolated from a marine-sediment fuel cell. Int. J. Syst. Evol. Microbiol..

[100] Ritalahti K.M., Justicia-Leon S.D., Cusick K.D., Ramos-Hernandez N., Rubin M., Dornbush J., Löffler F.E. (2012). *Sphaerochaeta globosa* gen. nov., sp. nov. and *Sphaerochaeta pleomorpha* sp. nov., free-living, spherical spirochaetes. Int. J. Syst. Evol. Microbiol..

[101] Gerritsen J., Hornung B., Staneva I., Ritarl J., Paulin L., Rijkers G., Schaap P.J., de Vos W.M., Smidt H. (2015). Comparative genomics and functional analysis of the genus Romboutsia provides insight into adaptation to an intestinal lifestyle. The Genus Romboutsia. Genomic and Functional Characterisation of Novel Bacteria Dedicated to Life in the Intestinal Tract.

[102] Sanchez-Andrea I., Sanz J.L., Stams A.J. (2014). Microbacter margulisiae gen. nov., sp. nov., a propionigenic bacterium isolated from sediments of an acid rock drainage pond. Int. J. Syst. Evol. Microbiol..

[103] Xue Y., Xu Y., Liu Y., Ma Y., Zhou P. (2001). *Thermoanaerobacter tengcongensis* sp. nov., a novel anaerobic, saccharolytic, thermophilic bacterium isolated from a hot spring in Tengcong, China. Int. J. Syst. Evol. Microbiol..

[104] Machi H.S., Ohashi A., Harada H., Hanada S., Kamagata Y., Sekiguchi Y. (2007). *Pelotomaculum propionicicum* sp. nov., an anaerobic, mesophilic, obligately syntrophic, propionate-oxidizing bacterium. Int. J. Syst. Evol. Microbiol..

[105] Chen S., Liu X., Don X. (2005). *Syntrophobacter sulfatireducens* sp. nov., a novel syntrophic, propionate-oxidizing bacterium isolated from UASB reactors. Int. J. Syst. Evol. Microbiol..

[106] Balch W.E., Schoberth S., Tanner R.S., Wolfe R.S. (1977). Acetobacterium, a new genus of hydrogen-oxidizing, carbon dioxide-reducing, anaerobic bacteria. Int. J. Syst. Evol. Microbiol..

