# Supplementary material for: Impact of Silver and Copper Oxide Nanoparticles on Anaerobic Digestion of Sludge and Bacterial Community Structure
Source: Nanomaterials (Basel). 2025 Feb 3;15(3):236. doi: 10.3390/nano15030236 (PMC11820454; doi:10.3390/nano15030236)
Supplement: Supplementary file 1 [file nanomaterials-15-00236-s001.zip › nanomaterials-3431872-supplementary.pdf]

Supplementary Material

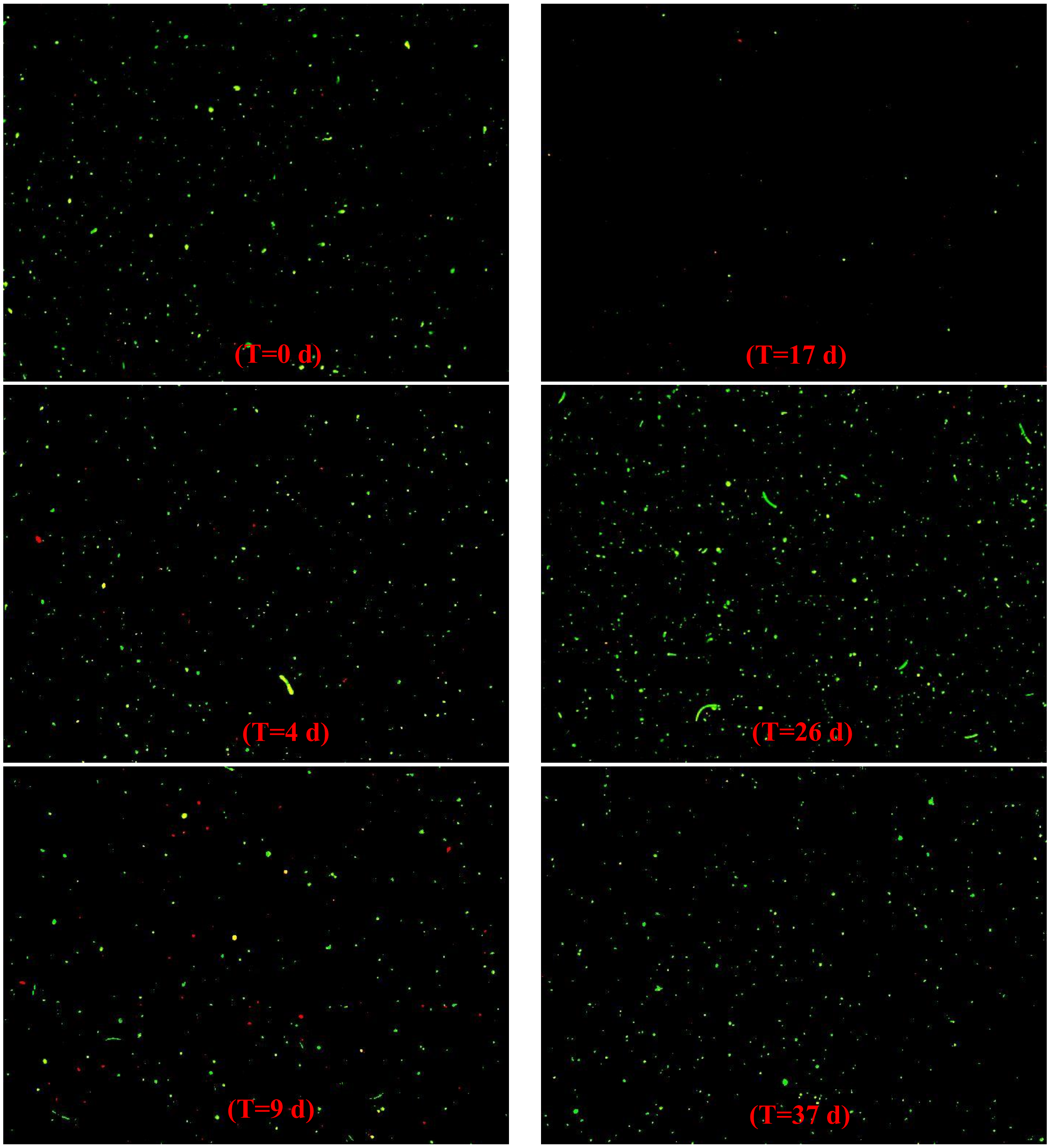

Figure S1. Live/Dead cells images for the control over time.

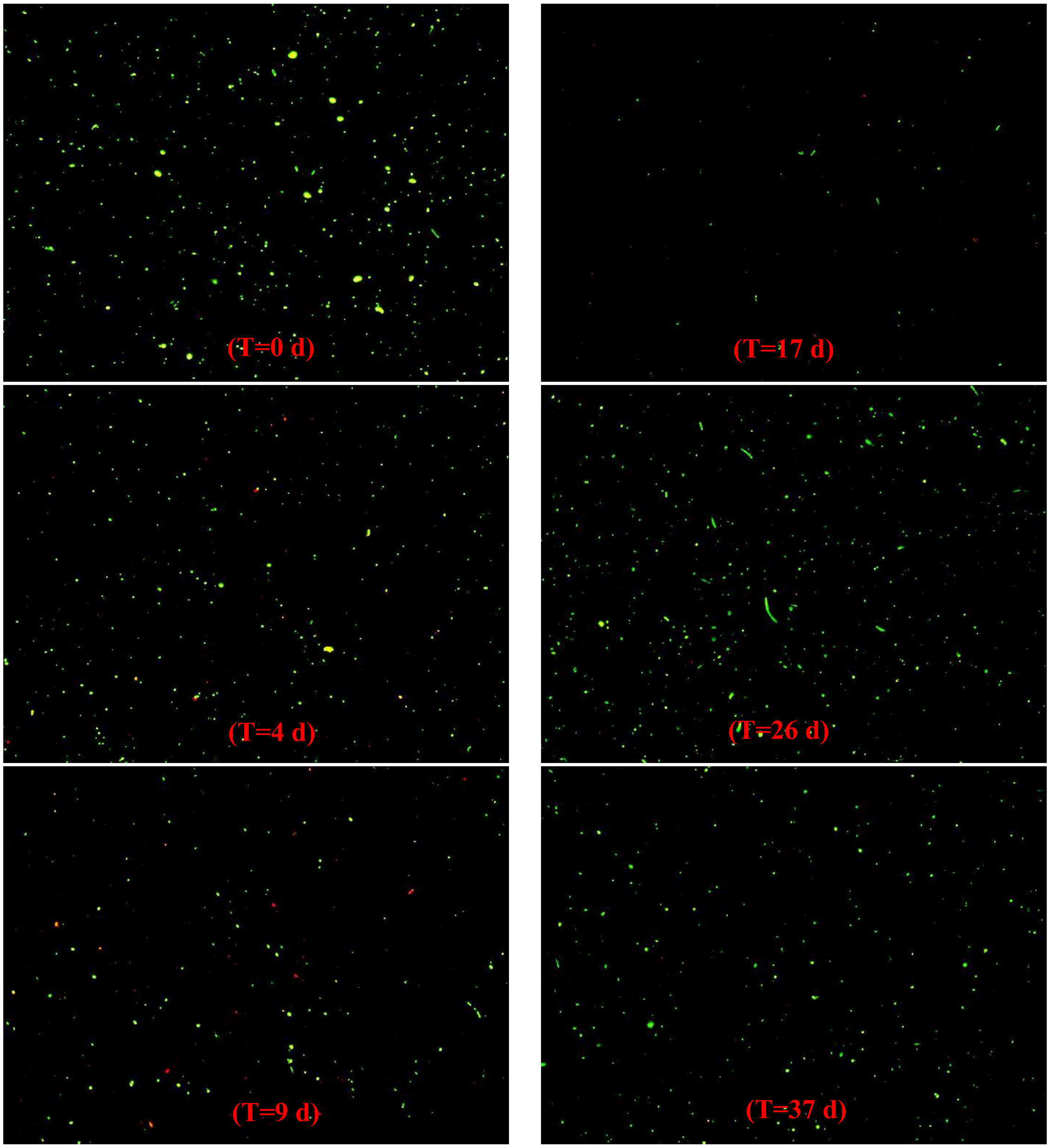

Figure S2. Live/Dead cells images for reactor A (2 mg AgNPs/ g TS of sludge).

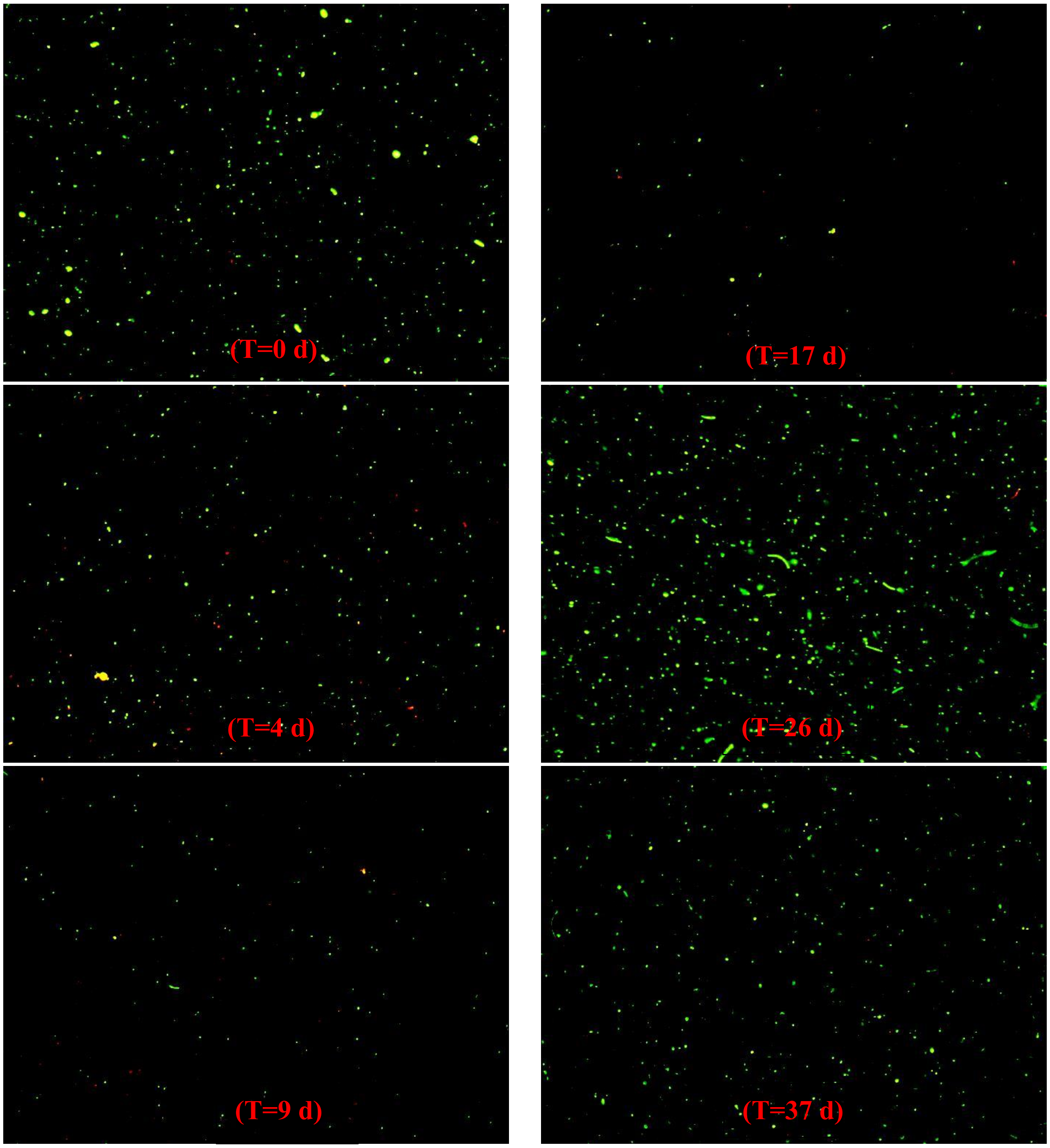

Figure S3. Live/Dead cells images for reactor B (10 mg AgNPs/ g TS of sludge).

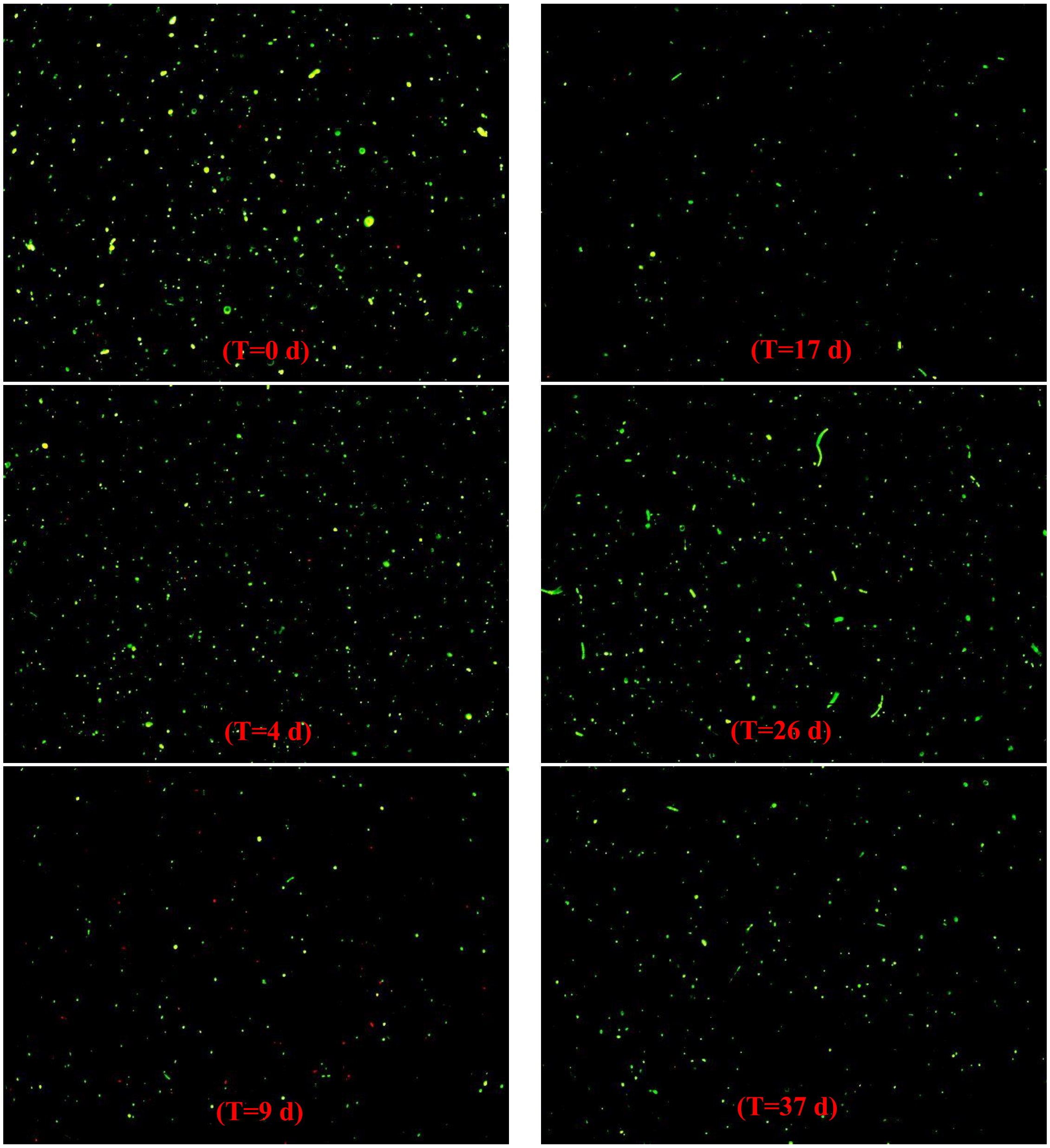

Figure S4. Live/Dead cells images for reactor C (30 mg AgNPs/ g TS of sludge).

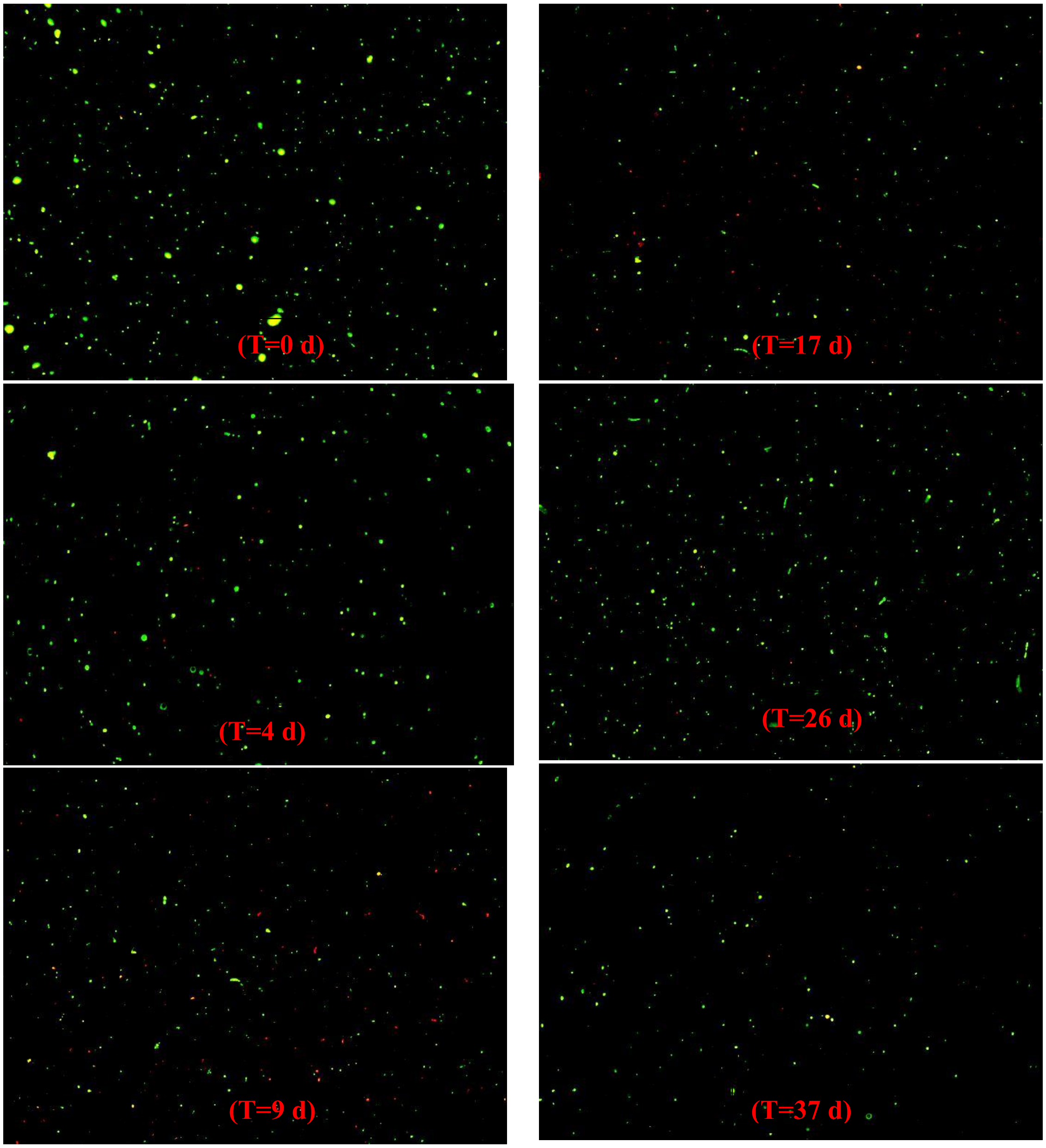

Figure S5. Live/Dead cells images for reactor D (2 mg AgNPs/ g TS of sludge).

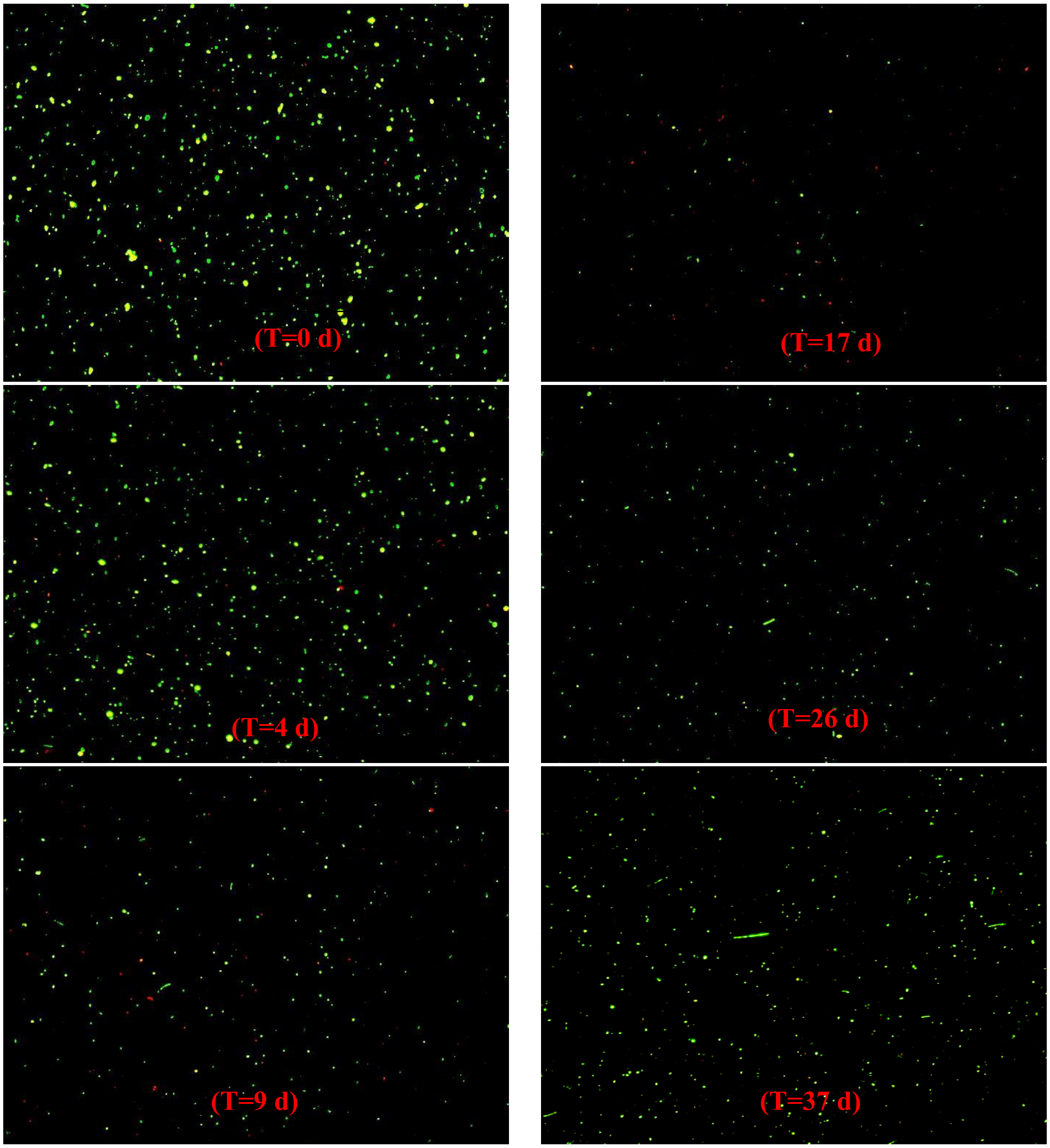

Figure S6. Live/Dead cells images for reactor E (10 mg AgNPs/ g TS of sludge).

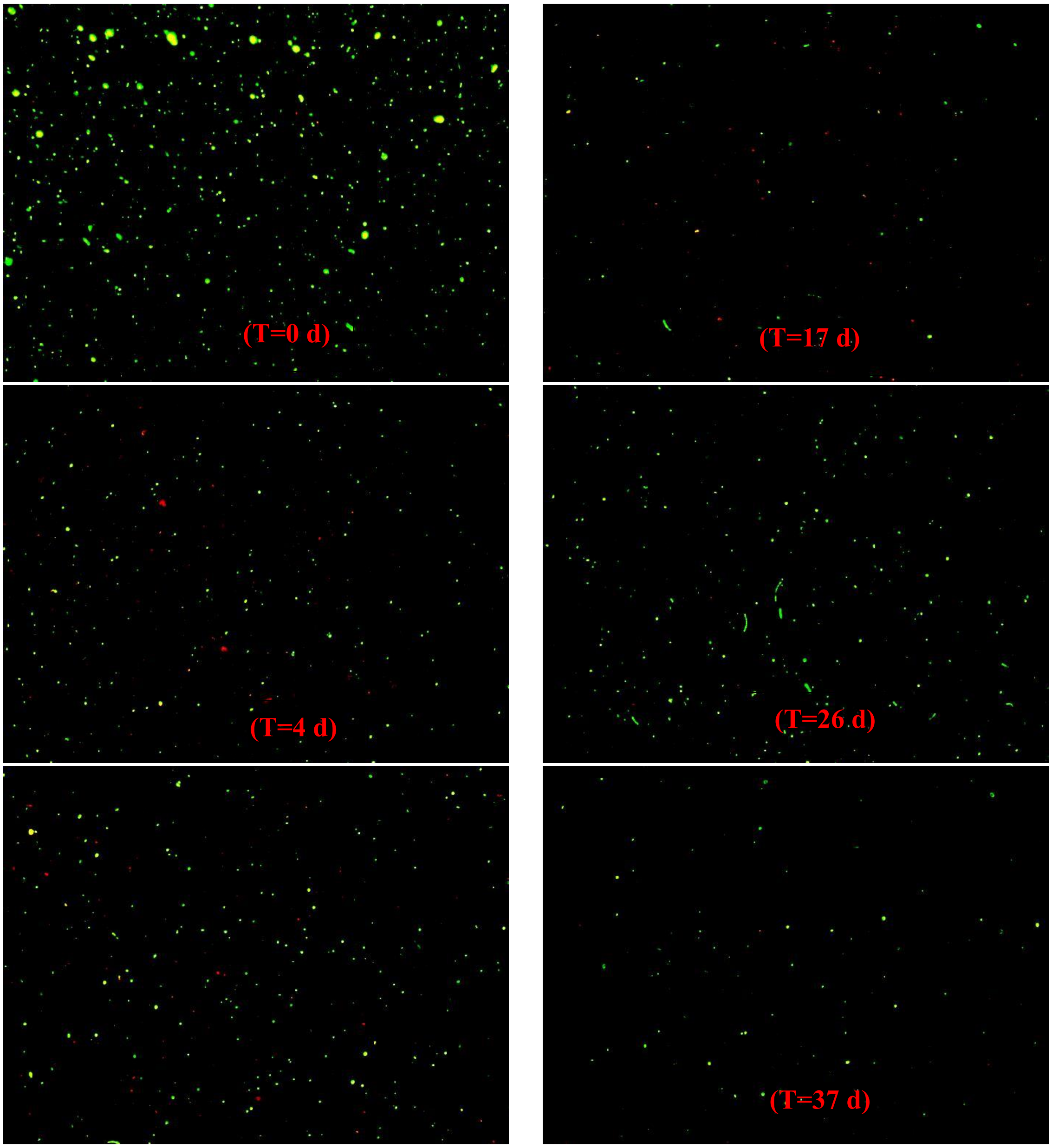

Figure S7. Live/Dead cells images for reactor F (30 mg AgNPs/ g TS of sludge).

Genus of interest and their important role in sludge anaerobic digestion

Table S1

Genus of interest and their role in sludge anaerobic digestion process

Genus	Specification	Function in AD	Action	Reference
Acidovorax	Gram-negative, motile or non-motile Aerobic/facultative anaerobic.	Utilize acetate for denitrification in activated sludge, nitrate reduction and fatty acids, sugars and proteins/amino acids assimilation.	Hydrolysis and acidogenesis.	[56,70]
Arcobacter	Gram-negative, motile, aerotolerance.	Denitrification and H_2S-producing bacteria. Assimilation of organic, amino acids, fatty acids, sugars and proteins/amino acids.	Hydrolysis and acidogenesis.	[57,58]
Novosphingobium	Gram-negative, non-motile, facultative anaerobic.	Degrading a number of organic compounds, including aromatic hydrocarbons, polycyclic aromatic hydrocarbons (PAH) polychlorophenols and carbohydrates. Assimilate fatty acids, sugars and proteins/amino acids.	Hydrolysis.	[71]
Syntophus	Gram-negative, obligate anaerobes.	Acidogenesis and degradation of fatty acids and benzoate in syntrophic association with hydrogen-using microorganisms.	Acetogenesis.	[72]
Sedimentibacter	Gram-negative, obligate anaerobes.	Acidogenesis and degradation of fatty acids, amino acids and pyruvate proteins. Sugars are not fermented by members of the genus.	Acidogenesis and acetogenesis.	[73]
Thauera	Gram-negative, motile. Facultative anaerobes	Denitrification. Utilizing aromatic compounds, amino acids and organic substrates such as sugars, acetate, lactate, pyruvate and ethanol, while using nitrate, nitrite or oxygen as electron acceptor.	Hydrolysis and acidogenesis.	[74]
Oscillibacter	Gram-negative, obligate anaerobes and motile.	Acidogenesis. Assimilate sugars.	Hydrolysis and acidogenesis.	[75]
Ruminiclostridium	Gram-positive, anaerobic organism	Acidogenesis of sugars derived from cellulose and production of extracellular multi-enzymatic complexes called cellulosomes, which efficiently degrade the crystalline cellulose.	Hydrolysis and acidogenesis.	[76]
Simplicispira	Facultative anaerobes.	Are able to assimilate fatty acids, sugars and proteins/amino acids	Hydrolysis and acidogenesis.	[77]
Clostridium	Gram-positive, obligate anaerobes.	Acidogenesis. Production of acetic acid, lactic acid and/or ethanol, propanol or butanol.	Hydrolysis, acidogenesis and Acetogenesis.	[78]
Trichococcus	Gram-positive, facultative anaerobes.	Assimilate fatty acids and sugar.	Acidogenesis.	[79]
Bacteroides	Gram-negative, obligate anaerobes, motile or non-motile.	Producing various acids such as lactic acid, propionic acid, formic acid and acetic acid from carbohydrates	Hydrolysis.	[80]

Alistipes	Gram-negative, obligate anaerobes, motile.	Acidogenesis. Assimilation of glucose to produced succinic and acetic acids as end products.	Acidogenesis.	[81]
Rhodoferax	Gram-negative, facultative anaerobes, motile.	Assimilation of a range of different carbon sources, including amino acids, sugars and short chain fatty acids.	Hydrolysis and acidogenesis.	[82]
Aeromonas	Gram-negative, facultative anaerobic.	Acidogenesis of organic matter and sugars.	Hydrolysis and acidogenesis.	[83]
Desulfotomaculum	Gram-positive, obligate anaerobes.	Sulfate-reducing bacteria utilizing sulfate (SO_4^{2-}) as terminal electron acceptor, reducing it to hydrogen sulfide (H_2S). Nitrogen fixation	Acetogenesis.	[84]
Lachnoclostridium	Gram-positive, obligate anaerobes.	Fermentative. Assimilate sugars. Acetate is the major end product of mono- and disaccharide acidogenesis.	Acidogenesis.	[85]
Hydrogenophaga	Gram-negative, anaerobic, facultative aerobic, motile.	Enhanced biological phosphorus removal. Denitrification, nitrate reduction using the oxidation of H_2 as an energy source and CO_2 as a carbon source.		[86]
Gracilibacter	Gram-negative, obligatorily anaerobic.	Acidogenesis. Acetate, lactate and ethanol are the main acidogenesis end products of glucose.	Acidogenesis.	[87]
Christensenella	Gram-negative, anaerobic, nonmotile.	Acidogenesis. Acetic acid and a small amount of butyric acid the end products of glucose acidogenesis.	Acidogenesis.	[88]
Moorella	Gram-positive, anaerobic.	Production of acetate by reducing carbon dioxide.	Acetogenesis.	[89]
Acidaminococcus	Gram-negative, anaerobes.	Assimilation of amino acids.	Acidogenesis.	[90]
Sunxiuqinia	Gram-negative, facultative anaerobic, nonmotile.	Assimilation of yeast extract, tryptone, casein and casamino acids with acidogenesis.	Hydrolysis.	[91]
Desulfovibrio	Gram-negative, obligate anaerobic, motile.	Acidogenesis. Sulfate-reducing bacteria.	Acidogenesis.	[92]
Syntrophomonas	Gram-negative, anaerobe, non-motile.	Fatty-acid-using bacterium, acetogen that require syntrophic interaction with H,-utilizing bacteria.	Acetogenesis.	[93]
Ruminococcus	Gram-positive bacterium, obligate anaerobic, non-motile.	Acetate and succinate are the major end products of cellulose and cellobiose acidogenesis.	Acidogenesis and acetogenesis.	[94]
Treponema	Gram-negative, anaerobic, motile.	End products of glucose acidogenesis are: lactic, acetic, and succinic acids, CO_2, and H.	Acidogenesis.	[95]
Caldicoprobacter	Gram-positive, obligate anaerobic.	Acidogenesis. Assimilation of sugars.	Acidogenesis.	[96]
Faecalicatena	Gram-positive, obligate anaerobe, motile.	Assimilation of sugars. Production of hydrogen sulfide.	Hydrolysis and acidogenesis.	[97]
Geobacter	Gram-negative, obligate anaerobe, motile.	Decomposition of organic compounds.	Hydrolysis and acidogenesis.	[98]
Prolixibacter	Gram-negative, facultative anaerobe, non-motile.	Acidogenesis of sugars.	Acidogenesis.	[99]

Sphaerochaeta	Gram-negative, anaerobe, non-motile.	Acidogenesis. Assimilation of fructose, galactose, glucose, mannose, raffinose, sucrose, xylose. Ethanol, acetate and formate are major end products.	Hydrolysis and acidogenesis.	[100]
Romboutsia	Gram-positive, obligate anaerobic, non-motile.	Acidogenesis of sugars, including glucose. Major end products are acetate, formate and sometimes lactate.	Hydrolysis, acidogenesis and acetogenesis.	[101]
Microbacter	Gram-negative, anaerobic, non-motile.	utilized yeast extract and various sugars as substrates and formed propionate, lactate and acetate as major acidogenesis products	Acidogenesis.	[102]
Thermoanaerobacter	Gram-negative, obligate anaerobic.	Assimilation of sugars and acetate is the main end product from glucose acidogenesis.	Acidogenesis and acetogenesis.	[103]
Pelotomaculum	Gram-positive, obligate anaerobic, non-motile.	Obligate syntrophic bacterium with methanogens. Anaerobic degradation of complex organic matter under methanogenic conditions.	Acetogenesis.	[104]
Syntrophobacter	Gram-negative, anaerobic, non-motile.	Degradation of complex organic matter under methanogenic conditions. The end products are acetate and CO_2.	Hydrolysis, acidogenesis and acetogenesis.	[105]
Acetobacterium	Gram-positive, anaerobic.	Acetogen.	Acetogenesis.	[106]
